# Intermittent Hormone Therapy Models Analysis and Bayesian Model Comparison for Prostate Cancer

**DOI:** 10.1007/s11538-021-00953-w

**Published:** 2021-11-19

**Authors:** S. Pasetto, H. Enderling, R. A. Gatenby, R. Brady-Nicholls

**Affiliations:** 1 Department of Integrated Mathematical Oncology, H. Lee Moffitt Cancer and Research Institute, 12902 Magnolia Drive, Tampa, FL 33612 USA; 2 Department of Radiation Oncology, H. Lee Moffitt Cancer and Research Institute, 12902 Magnolia Drive, Tampa, FL 33612 USA; 3 Department of Genitourinary Oncology, H. Lee Moffitt Cancer Center and Research Institute, 12902 Magnolia Drive, Tampa, FL 33612 USA; 4 Department of Radiology, H. Lee Moffitt Cancer and Research Institute, 12902 Magnolia Drive, Tampa, FL 33612 USA

**Keywords:** Prostate cancer, Intermittent hormone therapy models

## Abstract

The prostate is an exocrine gland of the male reproductive system dependent on androgens (testosterone and dihydrotestosterone) for development and maintenance. First-line therapy for prostate cancer includes androgen deprivation therapy (ADT), depriving both the normal and malignant prostate cells of androgens required for proliferation and survival. A significant problem with continuous ADT at the maximum tolerable dose is the insurgence of cancer cell resistance. In recent years, intermittent ADT has been proposed as an alternative to continuous ADT, limiting toxicities and delaying time-to-progression. Several mathematical models with different biological resistance mechanisms have been considered to simulate intermittent ADT response dynamics. We present a comparison between 13 of these intermittent dynamical models and assess their ability to describe prostate-specific antigen (PSA) dynamics. The models are calibrated to longitudinal PSA data from the Canadian Prospective Phase II Trial of intermittent ADT for locally advanced prostate cancer. We perform Bayesian inference and model analysis over the models’ space of parameters on- and off-treatment to determine each model’s strength and weakness in describing the patient-specific PSA dynamics. Additionally, we carry out a classical Bayesian model comparison on the models’ evidence to determine the models with the highest likelihood to simulate the clinically observed dynamics. Our analysis identifies several models with critical abilities to disentangle between relapsing and not relapsing patients, together with parameter intervals where the critical points’ basin of attraction might be exploited for clinical purposes. Finally, within the Bayesian model comparison framework, we identify the most compelling models in the description of the clinical data.

## Introduction

The prostate is an exocrine gland of most mammals’ male reproductive system. The normal prostate is dependent on androgens, specifically testosterone and 5*α*-dihydrotestosterone (DHT), for development and maintenance (Feldman and Feldman 2001). Prostate carcinoma (PCa) results from the abnormal growth of tissue from the prostate’s epithelial cells, which might induce metastasis in bones and lymph nodes. PCa is the second most common cancer in the USA and the second leading cause of cancer-related death after lung cancer (Siegel et al. 2021). The average male is 70 years of age at the time of diagnosis, with a strong of the distribution asymmetry biased toward older ages. PCa risk is often influenced by genetics. Men with a first-degree relative with PCa are twice as likely to develop it themselves; men with high blood pressure are also at higher risk of PCa. Treatment options typically include surgery, radiotherapy, high-intensity focused ultrasound, chemotherapy, and hormonal therapy.

Screening for PCa is commonly performed through rectal examination or the noninvasive blood biomarker prostate-specific antigen (PSA), although its efficiency remains controversial (Lin et al. 2008). Today, more robust marker indicators, such as the overexpression of prostate cancer gene 3 (PCA3) obtained from the messenger-RNA (mRNA) in the urines, are considered more suited to monitoring the cancer evolution (Bussemakers et al. 1999, p. 3; Laxman et al. 2008; Neves et al. 2008; Hessels and Schalken 2009, p. 3; Borros 2009; Qin et al. 2020). PSA is a measure of a hematic enzyme produced by the prostate. PSA levels between 4.0 and 6.5 µh L^−1^ are generally considered normal (with a strong dependence on age). PSA is naturally present in the serum, and usually, only a small amount of PSA of the prostate leaks into the blood. Hence, high levels are an indication of prostatic hyperplasia or cancer. Since prostate cells and their malignant counterparts require androgen stimulation to grow, prostate cancer can be treated by androgen deprivation therapy (ADT), a type of hormone therapy. This therapy reduces androgen-dependent (AD) cancer cells by preventing their growth and inducing cellular apoptosis.

Unfortunately, treating with ADT often results in a relapse in the form of hormone-refractory PCa due to the selection for the androgen-independent (AI) cells. Intermittent androgen deprivation (IAD) therapy, whereby treatment is cycled on and off, is often used as an alternative to ADT to delay treatment resistance. In IAD, androgen deprivation therapy is administered until a patient experiences a remission and then is withheld until the disease progresses up to a certain level. Clinical studies have shown that patients are responsive to multiple hormone therapy cycles, eventually delaying the androgen independence insurgence (Klotz et al. 1986; Larry Goldenberg et al. 1995; Bruchovsky et al. 2006).

We consider models of intermittent therapy due to clinical interest and solve the inference problem using longitudinal PSA data from the Canadian Prospective Phase II Trial of IAD for locally advanced prostate cancer. This work aims to present the first systematic comparative study of IAD models, emphasizing their ability to disentangle relapsing and not relapsing patients and compare the models in the Bayesian framework. The goal is to detect the single model (or the group of models) that best represent the information in the considered dataset and, therefore, if possible, the most promising biological frame representing them. A general and historical review of the available prostate cancer models can be found elsewhere (Phan et al. 2020).

In Sect. 2, we present the data included in our analysis. In Sect. 3, we introduce the statistical framework used to analyze the data. Section 4 presents an analysis of the models and their performance over the dataset utilizing the framework considered. Section 5 compares the performance of all the models, and Sect. 6 concludes and discusses the paper’s findings and future developments.

## Dataset

### Data Cohort

**Fig. 1:**
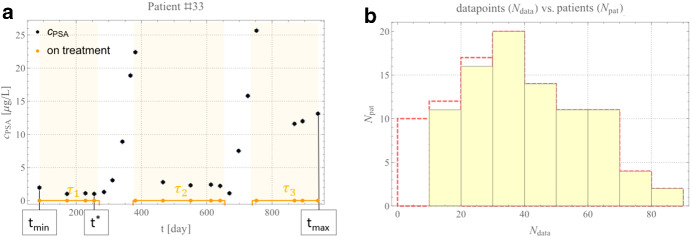
Model data. **a** PSA data for patient #33 from *t*_min_ = 88 [day] to t_max_ = 941 [day]. Black dots indicate PSA values (error bars are omitted due to little variability), orange points indicate where PSA was collected, and graphically represented as an orange continuous box function, evidenced only in this example panel by yellow shaded areas. Treatment intervals are labeled *τ*_1_, *τ*_2_ and *τ*_3_. t* is the first minimum of PSA in *τ*_1_. **b** Distribution of the number of data points per patient. The original data are shown by the red dashed lines, while the selected subset of patients used in this analysis is shown in the yellow shaded region (Color figure online)

We consider data from the Canadian Prospective Phase II Trial of intermittent ADT for biochemically recurrent prostate cancer (Bruchovsky et al. 2006, 2008). The total patient number is *N*_pat_ = 101. Their median pretreatment serum testosterone is 13.0 µg L^−1^, ranging between 0.4 and 23.0 µg L^−1^. Over a maximum of $n = 5$ intermittent ADT cycles, a median of 35.1–36.0 weeks is spent on-treatment (depending on *n*), and 25.6–53.7 weeks (e.g., *n* = 5 and *n* = 1 respectively) are off-treatment during the 6-year study. An example of a PSA profile for an individual patient is shown in Fig. 1a. This patient responded to treatment during the first two treatment cycles ($\tau_{1}$ and $\tau_{2}$) and progressed in his third cycle of treatment ($\tau_{3}$). The oscillatory dynamics demonstrate the effect of the intermittent treatment, with a decrease in PSA during treatment and an increase once treatment is turned off. Each data point is assigned with an error of 1 day in time (i.e., the time resolution of the dataset) and a maximal PSA error value $e_{{{\text{max}}}}$ assigned of $e_{{{\text{max}}}} = 0.1$ µg L^−1^ assumed from the literature (Borros 2009).

We set the minimal PSA detection threshold equal to ${\Delta }_{1} = 0.1$ µg L^−1^, i.e., any patient data below this threshold is set to $0.1$ µg L^−1^. Patients with a minimal per-day fluctuation below $2.0{ }$ µg L^−1^, i.e., a minimal per-day fluctuation of the KLK3 glycoprotein enzyme of PSA of a typical man (Morgentaler and Conners 2015), are excluded because such small fluctuations are considered natural and not pathological. To only consider PSA concentrations above Poisson noise, patients with less than ${\Delta }_{2} = \sqrt {N_{{{\text{pat}}}} }$ (i.e., the sample shot/Poisson noise) data points are also excluded. These exclusions result in our analysis considering data from 89 (*N*_pat_ = 89) rather than 101 patients. The patients’ distribution per number of data points used after the selection process is shown in Fig. 1b compared to the original distribution.

### Data Interpretation

The PSA trend shown in Fig. 1a is based on the interplay between cellular populations, i.e., with a compartment modeling approach. An androgen-dependent set of $N_{D} \ge 1$ cell populations (each with a concentration $n_{D,k} = n_{D,k} \left( t \right),$ $k = 1, \ldots ,N_{D}$ representing the compartment concentration [$\mu g L^{ - 1}$], $t \in {\mathbb{R}}$ time [day]) is assumed to contribute to the oscillatory behavior of PSA. Additionally, a set of time-dependent androgen-independent cellular populations of $N_{I} \ge 0$ cell populations $n_{I,l} = n_{I,l} \left( t \right)$, $l = 0, \ldots ,N_{I}$, also contributes to the PSA profile, such that PSA concentration $c_{\rm PSA}$ is given by $c_{\rm PSA} = f\left( {n_{D,k} ,n_{I,l} } \right),$ where $f \in C^{0}$ is a function belonging to the class of continuous solution $C^{0}$ (not necessarily smooth) of a suitably designed ODEs system$.$ Any further dependence on space, temperature, and pressure is generally neglected in the IAD models’ compartment approach. Furthermore, $f$ is often assumed to be a linear combination of the $N_{D}$ and $N_{I}$ compartments, e.g., $c_{\rm PSA} = \mathop \sum \limits_{k}^{{}} w_{k} n_{D,k} + \mathop \sum \limits_{l}^{{}} w_{l} n_{I,l}$ for some weights $w_{l}$ and $w_{k} .$

By assuming ADT to be highly effective in the first treatment interval $\tau_{1}$, we can set $n_{D} \left( {t \in \tau_{1} } \right) \cong 0$ for some *t* as an initial condition (hereafter i.c.). This approach does not necessarily hold for $\tau_{i}$ with $i > 1$: Generally, $\forall i$ where $c_{\rm PSA} \cong 0$, we can equally well assume this setting for the i.c. of the $n_{D,k} = n_{D,k} \left( t \right)$ equations. Equivalently we can assume that a non-holonomic (i.e., with inequalities) condition for the fitting procedure holds at the beginning of the patient time series $n_{D} \left( t \right) \le n_{I} \left( t \right)$ for some $t \in \tau_{1}$. Furthermore, in most of the models that we accounted for, these considerations are articulated with the addition of a few extra equations that interpret, at a local or global level in the parameter space, the contribution to $c_{{\textit{PSA}}} \left( t \right)$ by the androgen quota, cellular plasticity, staminal cells populations, or other model specificities.

Finally, we know from biological arguments that, under treatment, the models’ equations are designed to permit, at least for $c_{PSA}$, to tend asymptotically to the value $c_{\rm PSA} = 0$. Any model that does not permit the phase state $c_{\rm PSA} = c_{\rm PSA} \left( t \right)$ to reach approximatively null values for any $t$, i.e., ∄*t* ℝ: $c_{{{\text{PSA}}}} \left( t \right) \cong 0$, would fail to reproduce the patients whose first treatment is always successful (see Fig. 1a). We elaborate more on this in Sect. 4. Therefore, it is worth investigating if the models allow for stationary equilibria outside the treatment intervals and then if any of the patient best fit values have fallen close to those equilibria (when they exist). This behavior would imply a stationary or recurrent solution for the dynamics and, therefore, a constrained PSA’s evolution if this “basin of attraction” is achievable in a biological time of interest. We stress that this mathematical behavior does not imply that the patient can effectively reach the point of equilibrium on the biological timescale of interest or a plausible point regarding toxicity levels.

## Bayesian Inference

The Bayesian regression approach stems from the concept of probability as a measure of the plausibility of a model *given* the truth of the information in the data presented above. First, we encode the prior state of knowledge about the parameters considered ${\bf{p}} = \left\{ {p_{1} ,p_{2} ,\ldots} \right\}$ into a prior distribution function ${\bf{Pr}}\left( {{\text{p}}|I} \right)$, where $I$ represents any available information. Typically, this can be achieved with a flat, uniform, and not informative prior at the beginning or with a sharper prior when the model is better trained. We return to this point in Sect. 3.1. Secondly, we consider the dataset, $D$, through the likelihood $L\left( {{\bf{p}},D} \right) = {\text{Pr}}\left( {D|{\bf{p}},I} \right)$. Finally, the inference problem is solved, studying the probability distribution function encoding the knowledge of the prior and the information encoded in the likelihood of the data $\Pr \left( {\bf{p}}|{D,I} \right) \propto \Pr \left( {\bf{p}}|{I} \right)L\left( {\bf{p}},{D} \right)$.

Standard techniques to achieve this result are fully analytical (e.g., for some linear regression), approximated (e.g., asymptotic approximation, Laplacian approximation, Gaussian approximation, etc.), iterative (e.g., Levenberg–Marquardt), or fully numerical (e.g., simulated annealing genetic algorithms). The choice between these techniques depends on the nature of the problem. Here, we start using Laplacian approximation with hyperparameters (Hutter et al. 2011; Murphy 2012; Theodoridis 2015), as a few of the mathematical models that we consider herein are nested, to solve the inference problem (i.e., to search for the optimal set of parameters $\bf{p}$ that best represent the data). In order to confirm the inference results and to perform the Bayesian model comparison numerically, we test the results both against the nested sampling approach to the global likelihood (hereafter *evidence)* (Skilling 2004; Mukherjee et al. 2006; Feroz and Hobson 2008) and with the differential evolution search (Feoktistov 2006; Goode and Annin 2015) with up to aggressive scaling factors ($\le 0.9$) and cross probabilities ($\ge 0.1$). For the Bayesian model comparison part of our work, see Sect. 5, the nested sampling-based approach will embed the results in a natural framework.

Finally, we note that substantial limitations in the fitting procedure came from the sparse and irregular temporal sampling in the clinical data. This irregularity impacts the parameter space exploration due to the lack of condition on the PSA trend’s derivative. The partial derivative $\partial_{t} c_{PSA} \left( {{\bf{p}};t} \right)$ is not smooth, thus inhibiting using some straightforward optimization techniques based on the PSA curves’ gradients or convexity (Theodoridis 2015).

### The Priors $\Pr \left( {\bf{p}}|{I} \right)$

**Fig. 2:**
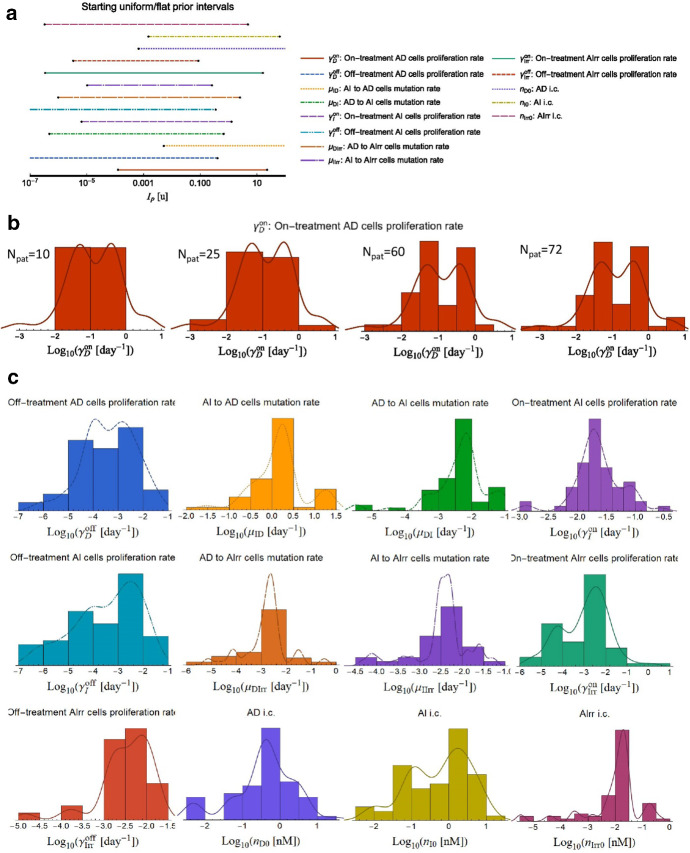
Model prior development. This example refers to the model by Hirata et al. 2010 and its 13 defining parameters. A similar technique is adopted for the other models. a. Initial bounded flat prior. b. Evolution of prior development for *γ*_*D*_^on^ [day^−1^] as the number of patients analyzed is increased (N_pat_ = {10,25,60,72}, respectively). **c** Final priors for the remaining 12 parameters (colors correspond to those shown in panel a) (Color figure online)

While robust approximations or numerical tools have been adopted for the Bayesian framework, special attention is paid to the use of priors. As mentioned, Bayesian inference requires the use of the priors, $\Pr \left( {\bf{p}}|{I} \right)$, for parameter estimation. With initially unknown priors, we implement uniform priors over the parameters’ full ranges (Fig. 2a). By requiring all model parameters to be positive, we can assume the Heaviside step function $\theta = \theta \left( {\bf{p}} \right)$ as (unnormalized) prior, this approach is generally referred to as “improper prior” as it is unbounded above, it cannot be normalized and therefore it does not have a mean, standard deviation, median, or quantiles. We set an upper bound for each parameter to be $p < p_{{{\text{max}}}}$ with a max value $p_{{{\text{max}}}} < + \infty$ $\forall p$ strictly. An alternative functional tested is the non-informative Jeffreys prior, ${\text{Pr}}\left( {{\bf{p}}|I} \right) \propto \sqrt {{\text{det}}\left( {{\text{F}}\left( {\bf{p}} \right)} \right)}$ with ${\text{F}}$ symbol referring to the Fisher Information matrix (Jeffreys 1946) and “det” to the matrix determinant.

Extra than testing with flat/Jeffreys priors, in the numerical nested sampling approach, we explore the parameter space logarithmically to avoid divergences, and once we reach a statistically significative sample, i.e., above the Poisson noise fluctuation ($\sim\sqrt {N_{{{\text{pat}}}} }$) shaping the posterior PDF, we proceed to implement the posterior as a prior for the patients analyzed in the dataset; finally, we reiterate by implementing a recursive determination of the prior (Fig. 2b, c). Further details can be found in Pasetto et al. (2021), where we discuss Bayesian analysis of retrospective data to guide clinical decisions.

## Analysis of IADT Mathematical Models

Here we consider only IAD models due to current clinical interest. Each model is presented and justified in a biological and mathematical sense in the original paper where the model was first presented, and we refer the reader to that papers for detailed model derivations. Similarly, the sensitivity analysis of the model parameters is presented in each paper individually, and we elaborate on it here only where necessary. We will refer to the relapsing patient set as ${\Omega }$-set and not relapsing to as relapse $\neg {\Omega }$-set.

We parametrize the individual IAD data with a patient-specific control function $T_{ps}$ defined as follows: $T_{ps} \left( t \right) = \sum\nolimits_{i = 1}^{n} {{\bf{1}}_{{\tau_{i} }} } \left( t \right)$, $0 < t \in \left[ {t_{{{\text{min}}}} ,t_{{{\text{max}}}} } \right]$^1^ with $t_{{{\text{min}}}}$ and $t_{{{\text{max}}}}$ minimum and maximum patient-specific treatment under consideration (e.g., Fig. 1a) and $t_{{{\text{min}}}}$ generally after the first treatment drop; $n \ge 1$ is the number of intervals $\tau_{i}$ considered. $\tau_{i} \subseteq \left] {t_{{{\text{min}}}} ,t_{{{\text{max}}}} } \right[\forall i$ is referred to as the “i^th^ treatment cycle,” and ${\bf{1}}_{{\tau_{i} }}$ is the indicator function^2^ for the interval $\tau_{i}$ (defined as ${\bf{1}}_{{\tau_{i} }} = 1$ for $t \in \tau_{i}$, 0 otherwise). For modeling purposes, the weights/errors, $e_{i}$ for each data $i$, have been assigned either uniformly $e_{i} = {\text{cnst}}.\forall i$ or with a linear decreasing relevance from the last PSA concentration $c_{PSA}$ peak, say $\hat{c}_{PSA}$ (e.g., in $\tau_{3}$ of Fig. 1) with $e_{i} = \left| {c_{{{\text{PSA}}i}} - \hat{c}_{PSA} } \right|_{{t = \hat{t}}}$, i.e. at $t = \hat{t}$, and $e_{i} = \left| {\hat{c}_{{{\text{PSA}}}} } \right| - \left| {\hat{t} - t} \right| + \left| {c_{{{\text{PSA}}i}} } \right|$ for $t \ne \hat{t}$. Finally, we performed sensitivity analysis on all the models included here. Comments on the technique adopted are technical and left to Supplement A.

### Ideta et al. (2008)

The model by Jackson (2004) can be considered the continuous ADT model prototype. Its extension to IAD therapy of interest was presented by Ideta et al. (2008). In this model (hereafter, I08), the authors drop the dependence of Jackson’s model on the spatial distribution, which is only of theoretical interest but not resolved in clinical PSA data. Model simulations predict that intermittent ADT can only prevent progression if normal androgen levels decrease the growth rate of AI cells, which may be biologically unlikely since AI cells have androgen receptors with increased sensitivity (Grossmann et al. 2001). We consider the I08 model in the following form:

1 $$\begin{array}{*{20}l} {\frac{{{\text{d}}n_{D} }}{{{\text{d}}t}} = \left( {\gamma_{D} - \delta_{D} - \mu_{{\textit{{\textit{DI}}}}} } \right)n_{D} ,} \hfill \\ {\frac{{{\text{d}}n_{I} }}{{{\text{d}}t}} = \mu_{{\textit{{\textit{DI}}}}} n_{D} + \left( {\gamma_{I} - \delta_{I} } \right)n_{I} ,} \hfill \\ \end{array}$$

with initial conditions $n_{D} \left( {t_{0D} } \right) = n_{D0} ,n_{I} \left( {t_{0I} } \right) = n_{I0}$.^3^ As previously mentioned, $n_{D}$ and $n_{I}$ are the androgen-dependent and -independent population number of cells (or concentration). $\gamma_{i}$ and $\delta_{i} , i \in \left\{ {D,I} \right\}$ are growth and apoptosis rates for AD and AI cells, given, respectively, by:

2 $$\begin{array}{*{20}l} {\gamma_{D} = \gamma_{D\max } \left( {\gamma_{DA} + \left( {1 - \gamma_{DA} } \right)\frac{{c_{A} }}{{c_{A} + k_{DA\gamma } }}} \right),} \hfill & {\gamma_{I} = 1 - \left( {1 - \frac{{\delta_{IA} }}{{\gamma_{IA} }}} \right)\frac{{c_{A} }}{{c_{A0} }},} \hfill \\ {\delta_{D} = \delta_{D\max } \left( {\delta_{DA} + \left( {1 - \delta_{DA} } \right)\frac{{c_{A} }}{{c_{A} + k_{DA\delta } }}} \right),} \hfill & {\delta_{I} = 1.} \hfill \\ \end{array}$$

In Eq. (2) $\gamma_{{D{\text{max}}}}$ and $\delta_{{D{\text{max}}}}$ are the maximal AD proliferation and apoptosis rates, $\delta_{DA}$ is a control parameter on the effect of low androgen levels on the AD apoptosis rate, $k_{DA\gamma } \ne 0$ is the AD half-saturation rate, $k_{DA\delta } \ne 0$ is the AD apoptosis rate dependence on androgen. Finally, $\delta_{IA}$ and $\gamma_{IA} \ne 0$ modulate hormonally patient failing death and growth. Mutation from AD to AI cells are allowed at a mutation rate:

3 $$\mu_{{\textit{{\textit{DI}}}}} = \mu_{{{\textit{{\textit{DI}}}}{\text{max}}}} \left( {1 - \frac{{c_{A} }}{{c_{A0} }}} \right),$$

thus, the mutation rate decreases as the androgen (here normalized at its homeostatic level $c_{A0} \ne 0$) approaches its max value $\mu_{{{\textit{{\textit{DI}}}}{\text{max}}}}$. A decoupled ODE model of the serum androgen concentration under treatment $c_{A}$ is given by:

4 $$\frac{{{\text{d}}c_{A} }}{{{\text{d}}t}} = \delta_{cA} \left( {c_{A0} - c_{A} } \right) - \delta_{cA} c_{A0} T_{ps} ,$$

with initial condition $c_{A} \left( {t_{{0{\text{A}}}} } \right) = c_{A0} \ne 0,$ where $\delta_{cA}$ is the androgen clearance rate. Here $T_{ps} = T_{ps} \left( t \right)$ is the patient treatment-specific function as defined in Sect. 2.1. Finally, the PSA density concentration of interest to us, $c_{PSA}$, is a linear combination with weight $w_{i}$ of the population densities

5 $$c_{{{\text{PSA}}}} = \mathop \sum \limits_{{i \in \left\{ {D,I} \right\}}}^{{}} w_{i} n_{i} .$$

Based on the original analysis of Ideta et al. and the available dataset, we explored two versions of this model, namely, where $\delta_{IA} = \gamma_{IA}$ in Eq. (2), i.e., $\gamma_{I} = {\text{cnst}}.$ (hereafter model I08A) and the original form of the equations ($\delta_{IA} \ne \gamma_{IA}$ hereafter model I08B).

#### I08A in the Context of the Data

We noted that the system of equations (hereafter SoE) composed by Eqs. (1)–(4) decouples in the androgen concentration $c_{A}$. The analysis of the system results in a line of infinite equilibria on the intersection of the plane $n_{D} = 0$ with the plane $c_{A} = c_{{{\text{A}}0}} - c_{{{\text{A}}0}} T_{ps}$ in the space of phase-state variables $\left( {n_{D} ,n_{I} ,c_{A} } \right)$. Thus, $c_{A} = c_{{{\text{A}}0}}$ off-treatment and $c_{A} = 0$ on-treatment. Standard linear stability analysis (Wiggins 2003) shows that the Jacobian of the system produces a null generalized eigenvalue $\lambda_{1} = 0$, a negative one $\lambda_{2} = - \delta_{A}$, and a more complicate third generalized eigenvalue that takes, off-treatment, the elegant form:${ }\lambda_{3}^{{{\text{off}}}} = \gamma_{{{\text{Dmax}}}} + \frac{{\left( {\gamma_{{{\text{DA}}}} - 1} \right)\gamma_{{{\text{Dmax}}}} k_{{{\text{D}}\gamma /2}} }}{{c_{{{\text{A}}0}} + k_{{{\text{D}}\gamma /2}} }} - \delta_{{{\text{Dmax}}}} - \frac{{\left( {\delta_{{{\text{DA}}}} - 1} \right)\delta_{{{\text{Dmax}}}} k_{{{\text{D}}\delta /2}} }}{{c_{{{\text{A}}0}} + k_{{{\text{D}}\delta /2}} }}.$ The sign of $\lambda_{3}^{{{\text{off}}}}$ can be evaluated for the best-fit parameter values that result from the inference works of Sect. 3 in the patients’ cohort considered here (Sect. 2), resulting in being always positive for all the patients. Therefore, the above-found equilibria lines represent a 1D nonstable manifold, and further investigations (e.g., in the context of the central manifold theory) are not of additional interest to us.

We are indeed more interested to further exploit the characteristics of the present dataset in the context of this model by using the decoupled nature of the serum androgen concentration $c_{A}$. All the patients are considered from their first cycle of treatment, starting with $T_{ps} \left( t \right) = 1$ for $t \in \tau_{1}$. Hence, we can emulate with a Heaviside step function $T_{ps} = \theta \left( { - t} \right)$ a cycle of treatment followed by the off-treatment period for a suitable cyclic interval (on–off, on–off, on–off, and so forth) around the off-treatment start, set at $t = 0$. Within this approach, the general solution of Eq. (4) is algebraic and reads:

6 $$c_{A} \left( t \right) = c_{{{\text{A}}0}} e^{{ - \delta_{A} \left( {t + 1} \right)}} \left( {e^{{\delta_{A} }} \theta \left( {e^{{\delta_{A} t}} - 1} \right) + 1} \right).$$

**Fig. 3:**
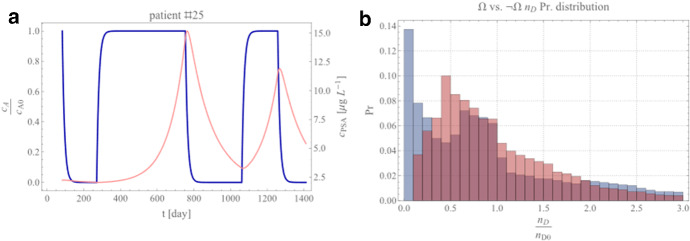
Ideta et al. model analysis results. **a** Evolution of the normalized androgen concentration *c*_A_ (normalized to its homeostatic value *c*_A0_, (left y-axis) as a function of time (blue curve) for a representative patient. For completeness, the PSA profile is also reported in light red (right y-axis). **b** Normalized androgen-dependent probability distribution functions on the cohort of best-fit parameters for progressive ($\Omega$, red) and responsive ($\neg \Omega$ , blue) sets (Color figure online)

This equation is monotonic on the two phases on/off-treatment because the derivative $dc_{A} /dt = c_{{{\text{A}}0}} \delta_{A} e^{{ - \delta_{A} \left( {t + 1} \right)}} \left( {e^{{\delta_{A} }} \theta - 1} \right)$ is never null neither for $t < 0$, i.e., on-treatment nor for $t \ge 0$, off-treatment. By splitting the treatment in on/off-time, we can always reverse the bilinear map $c_{A} = c_{A} \left( t \right)$ in $t = t\left( {c_{A} } \right)$. For example, in our case, it reads $t = - \frac{1}{{\delta_{A} }}{\text{log}}\left( {\frac{{c_{A} }}{{c_{A0} }}} \right) + \delta_{A}$ on-treatment and $t = \frac{1}{{\delta_{A} }}{\text{log}}\frac{{c_{A0} \left( {{\text{e}}^{{ - \delta_{A} }} - 1} \right)}}{{c_{A} - c_{A0} }}$ off-treatment^4^ for $c_{A} \ne c_{A0} ,$ and $c_{A} \ne 0$ and $\delta_{A} \ne 0$. We exploit Eq. (6) to obtain the probability distribution function (PDF) of the orbits over all the sets of patients remapping each cycle over the phase space section $\left( {O,n_{D} ,n_{I} } \right)$. We take advantage by the sharp $c_{A}$ passage from its homeostasis value $c_{A0}$ to null and vice versa in conjunction with the bijection map just found. Figure 3a shows the $c_{A}$ profile for a representative patient. While time is a monotonic increasing function, the map we considering is one-to-one only over the treatment cycle $T_{ps} = 1$ and the off-cycle $T_{ps} = 0$, respectively, and in these two tracks we can write the SoE as $n_{D} = n_{D} \left( {t\left( {c_{A} } \right)} \right) = n_{D} \left( {c_{A} } \right)$ and $n_{I} = n_{I} \left( {c_{A} } \right)$. As $c_{A}$ sharply switches from $c_{A} = c_{A0}$ and $c_{A} = 0$, we can limit ourselves to a first-order solution of the SoE. After simple algebra, we arrive at the approximate solution of the SoE in the form:

7 $$\begin{array}{*{20}l} {n_{D} \simeq n_{D0} - \frac{1}{{c_{A0} \delta_{A} }}n_{D0} \left( {c_{A} - c_{A0} } \right)\left( {\frac{{\gamma_{D\max } \left( {c_{A0} + \gamma_{A} k_{D\gamma /2} } \right)}}{{c_{A0} + k_{D\gamma /2} }} - \frac{{\delta_{D\max } \left( {c_{A0} + \delta_{D} k_{D\delta /2} } \right)}}{{c_{A0} + k_{D\delta /2} }}} \right),} \hfill \\ {n_{I} \simeq n_{I0} ,} \hfill \\ \end{array}$$

to the first order in $c_{A}$ (and where $\simeq$ means asymptotic-to). As evident, the second equation remains close to its initial value $n_{I0}$, while the first is perturbed away, suggesting that we can first sample the PDF of the dataset for fixed values in $n_{I}$ around $n_{I0}$ and then investigate the PDF as sampled from the best fit obtained by the patient in the trial with Eq. (7). The results are shown in Fig. 3b. The trend of the two distributions for the development of resistance and continuing response patients is comparable as above the starting value $n_{D} = n_{D0}$, while the trend diverges for smaller values $n_{D}$. Because we assume $n_{D}$ is a proxy for $c_{PSA}$ at small values of $n_{I}$, as evicted from Eqs. (5) and (7), if the model correctly interprets the data, then *a patient with* an initial *PSA-drop below 10% of its initial value* is highly likely to be a continuous responder. The risk of resistance development grows to about *50%* when the initial drop in PSA is around *30*%.

#### I08B in the Context of the Data

For I08B, where $\delta_{IA} \ne \gamma_{IA}$, the ratio presented in Eq. (3) evidences the structural non-identifiability of the SoE. The treatment of the equilibria and their stability is more straightforward in this model form than in I08A. The only equilibrium point is given by $\{ n_{D} ,n_{I} ,c_{A} \}_{{{\text{eq}}}} = \left\{ {0,0,c_{{{\text{A}}0}} - c_{{{\text{A}}0}} T_{ps} } \right\}$ with generalized eigenvalues $\lambda_{i}$ of the Jacobian at the equilibrium given by $\lambda_{1} = \left( {T_{ps} - 1} \right)\left( {\gamma_{{{\text{IA}}}} - \delta_{{{\text{IA}}}} } \right)\gamma_{IA}^{ - 1}$ with $\gamma_{IA} \ne 0$, $\lambda_{2} = \lambda_{2}^{{{\text{I}}08{\text{A}}}}$ and $\lambda_{3} = \lambda_{3}^{{{\text{I}}08{\text{A}}}}$. Following the I08A assumptions, we investigate the model under the conditions $\delta_{{D{\text{max}}}} > \gamma_{{D{\text{max}}}}$, $\delta_{DA} > 1$, and by requiring that $\mu_{{{\text{max}}}} < \gamma_{I} - \delta_{I}$ to avoid the annihilation of the populations. Under these conditions, we can prove that $\lambda_{1} \le 0$ and $\lambda_{2} \le 0$, and that for $\lambda_{3}$ it holds the same consideration as for I08A due to the non-stable nature of the resulting equilibrium manifold.

Analogous consideration on the non/relapsing treatment holds for I08B as for I08A, but with more straightforward treatment for I08B than for I08A: the two equilibria at homeostasis $c_{A} = c_{A0}$ and at null androgen concentration, $c_{A} = 0$, attract the dynamics as for I08A and self-explain the orbit profiles. Therefore, the identical results from the inference of I08B on patients’ trials can be obtained for the PDF but are not depicted again.

### Eikenberry et al. (2010)

The model developed by Eikenberry et al. (2010, hereafter E10) was an attempt to describe the interaction between testosterone (T, the primary androgen in the serum), its enzyme 5a-reductase to dihydrotestosterone (DHT), and their binding (T:AR and DHT:AR) with the androgen receptors (AR) in the prostate. Because of model E10’s versatility, we have included it in the IAD treatment model comparison. Of note, the authors have not proposed the model to fit data, and here we reinterpret E10 beyond the scope of the original paper. The modulation due to intermittent IAD is assumed in testosterone time modulation. While a linear relation might not be readily available from the literature between testosterone and PSA level (Elzanaty et al. 2017), we recode the testosterone concentration $n_{T}$, in E10 as follows:

8 $$\frac{{{\text{d}}n_{T} }}{{{\text{d}}t}} = n_{T} \left( {\delta_{T} - \frac{{\mu_{cat} n_{5\alpha } }}{{k_{M} + n_{T} }} - \kappa_{T:R} n_{R} } \right) + \delta_{T:R} q_{T:R} - \left( {T_{ps} - 1} \right)\Upsilon \left( {n_{S} } \right),$$

which we couple with the original system of equations:

9 $$\begin{gathered} \frac{{{\text{d}}n_{R} }}{{{\text{d}}t}} = n_{R} \left( {\gamma_{R} - \delta_{R} - \kappa_{DHT} n_{DHT} - \kappa_{T:R} n_{T} } \right) + \delta_{DHT:R} q_{DHT:R} + \delta_{T:R} q_{T:R} , \hfill \\ \frac{{{\text{d}}n_{DHT} }}{{{\text{d}}t}} = \frac{{\mu_{cat} n_{5\alpha } n_{T} }}{{k_{M} + n_{T} }} - n_{DHT} \left( {\delta_{DHT} + \kappa_{DHT} n_{R} } \right) + \delta_{DHT:R} q_{DHT:R} , \hfill \\ \frac{{{\text{d}}q_{T:R} }}{{{\text{d}}t}} = \kappa_{T:R} n_{R} n_{T} - \delta_{T:R} q_{T:R} , \hfill \\ \frac{{{\text{d}}q_{DHT:R} }}{{{\text{d}}t}} = \kappa_{DHT} n_{DHT} n_{R} - \delta_{DHT:R} q_{DHT:R} , \hfill \\ \end{gathered}$$

with five nominals initial conditions: $n_{R0} = n_{R} \left( {t_{0R} } \right)$, $n_{T0} = n_{T} \left( {t_{0T} } \right)$, $n_{DHT0} = n_{DHT} \left( {t_{0DHT} } \right)$, $q_{T:R0} = q_{T:R} \left( {t_{0T:R} } \right)$ and $q_{DHT:R0} = q_{DHT:R} \left( {t_{0DHT:R} } \right)$. Here, the treatment function $T_{ps}$ modulates testosterone influx into the prostate-function $\Upsilon \left( {n_{S} } \right)$ original in E10 and that we are going to adopt here, where $n_{{\text{S}}}$ is the testosterone serum concentration. Furthermore, we consider the androgen receptor concentration $n_{R}$ and the dihydrotestosterone concentration $n_{DHT}$ together with two quota concentrations $q_{T:R}$ and $q_{DHT:R}$ (Droop 1968), here, taken to be the T:AR complex and the DHT:AR complex concentration, respectively. $\gamma_{R}$ is the AR production rate, $\delta_{R}$ is the AR degradation rate, $\delta_{T}$ is the testosterone-specific degradation rate, and $\delta_{DHT}$ is the dihydrotestosterone degradation rate. The mass-action constants for the androgen-dependent component (testosterone) and dihydrotestosterone binding the AR are $\left\{ {\kappa_{a}^{T} ,\kappa_{d}^{T} ,\kappa_{a}^{{{\text{DHT}}}} ,\kappa_{d}^{{{\text{DHT}}}} } \right\}$, and the $5\alpha$ reductase converts T to DHT by Michaelis–Menten enzyme kinetics with concentration $n_{5\alpha }$, turnover number $\mu_{cat}$ and constant $k_{M} \ne 0$.

#### The Model in the Context of the Data

If we set $a \equiv \mu_{{{\text{cat}}}} n_{5\alpha } - \delta_{T} k_{M}$, $b \equiv \left( {1 - T_{ps} } \right)\Upsilon \left( {n_{s} } \right)$, and ${\Delta } \equiv \sqrt {(a + b)^{2} - 4b\delta_{T} k_{M} }$, then two critical points can be isolated at the intersection of the nullclines hyperplanes of the phase space. On-treatment, the first point $\left\{ {n_{R} ,n_{T} ,n_{DHT} ,q_{T:R} ,q_{DHT:R} } \right\}_{\text{eq}}^{{\left( {1,2} \right)}} = \left\{ {0,0,0,\frac{ - a - b \mp \Delta }{{2\delta_{T} }},\frac{ - a + b \pm \Delta }{{2\delta_{DHT} }}} \right\}$ holds as soon as $\mp a + b + {\Delta } \le 0 \wedge \mp a + {\Delta } \le b$. While only the second of these equilibria is of biological interest, it is not a stable equilibrium. Obtaining the complete set of generalized eigenvalues requires a cumbersome solution of three cubic equations, yet the check for the stability requires much less effort once we realize that one of the generalized eigenvalues from the characteristic equations reads simply $\delta_{T} - \frac{{4\delta_{T}^{2} \mu_{{{\text{cat}}}} k_{M} n_{5\alpha } }}{{(a + b - \Delta - 2\delta_{T} k_{M} )^{2} }}$ where $a + b - {\Delta } - 2\delta_{T} k_{M} \ne 0$ and that it proves to be always positive for all the inference results in the trial patients.

**Fig. 4:**
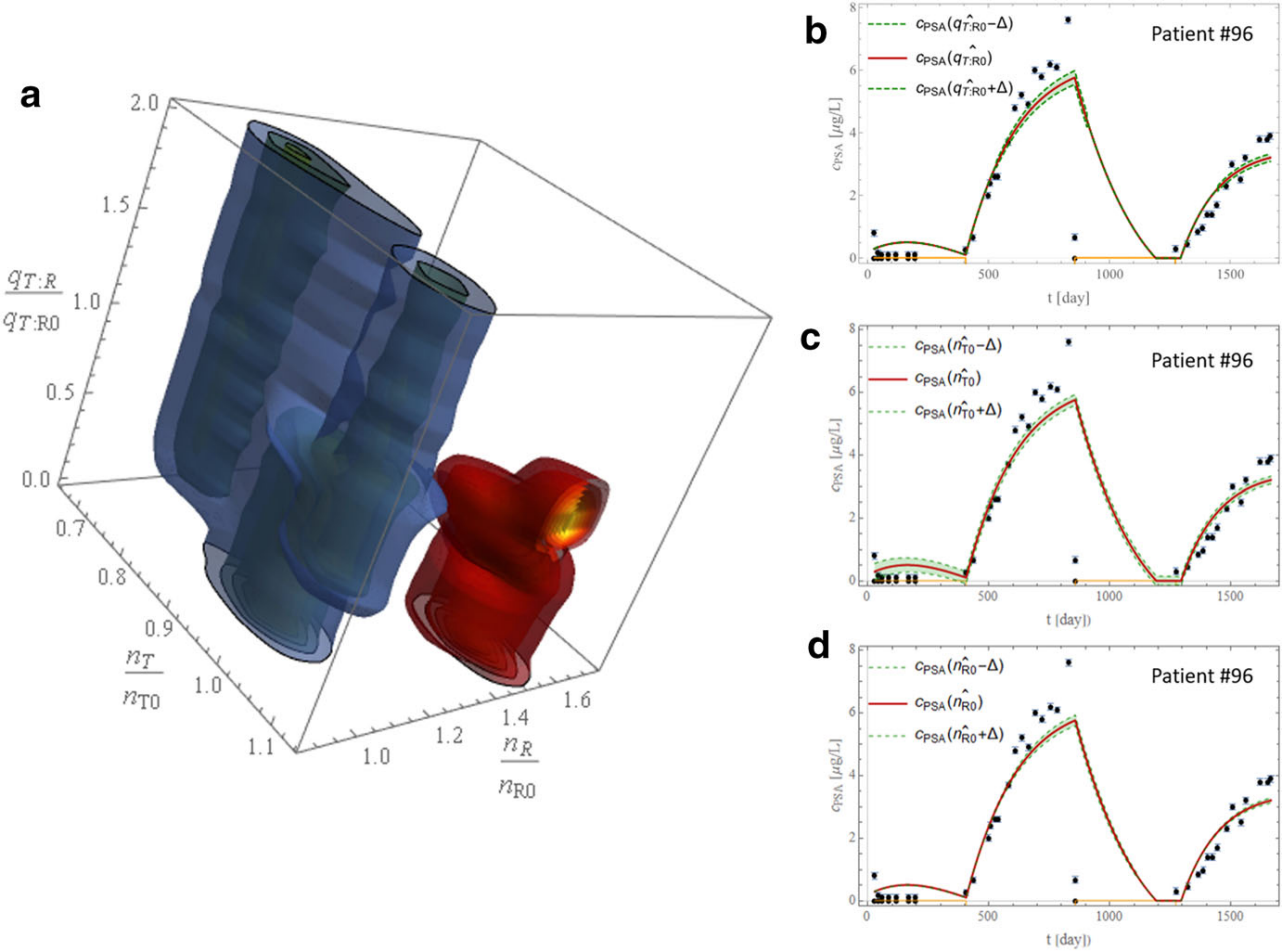
Eikenberry et al. model analysis results. **a** Probability distribution function of the $q_{T:R}$, *n*_T_, and *n*_R_ space. The isocontours for the Pr of $\Omega$ and $\neg \Omega$ sets are shown in blue and red, respectively. A few isocontours are shown at the border-slicing-planes for Pr = {0.1,0.68,0.95}. **b**–**d** Sensitivity of *c*_PSA_ in response to changes in the normalized values of $q_{T:R}$, *n*_T_ and *n*_R_ for a representative patient. The optimal fit *c*_PSA_ dynamics (i.e., for optimal parameters $\widehat{\bf{p}}$) and corresponding data are shown by the red curve and black dots with error bars, respectively; dashed green curves and the corresponding shadows show the sensitivity of *c*_PSA_ when the parameters are increased/decreased by Δ (Color figure online)

Finally, we note how the model could represent an essential instrument for investigating the relapsing mechanism evidenced in some patients, which remains one of the goals of this work for its potential clinical implications. We identify three over five state variables by inspecting the phase-state space with a striking separation between ${\Omega }$ and $\neg {\Omega }$. Figure 4a shows the 3D probability distribution function of $n_{T} ,n_{R} ,$ and $q_{T:R}$. The density map of the temporal evolution of ${\Omega }$ and $\neg {\Omega }$ sets clusters (over the orbital evolution spanned by the patients analyzed) on a well distinct area of the phase space, splitting in the $n_{T}$ vs. $n_{R}$ space and at least partially in the orthogonal $q_{T:R}$ space.

In Fig. 4, panels b, c, and d, we exploited the Direct Differential Method (DDM) for sensitivity analysis to track the time dependence of the sensitivity $S_{c_{{\textit{PSA}}}j} \equiv \frac{{\partial c_{PSA} \left( {t,\hat{\bf{p}}} \right)}}{{\partial p_{j} }}$ computed at the best fit parameter values $\hat{\bf{p}}$, where $\bf{p} = \left\{ {n_{T0} ,n_{R0} ,q_{T:R0} } \right\}$, respectively. As shown in Fig. 4b–d, a slight variation of the parameters does not dramatically affect the trend of $c_{PSA} .$ Thus, there is minimal sensitivity of $c_{PSA}$ to the parameters. This result shows that *the PDF of the combination of parameters investigated might be an excellent tool to explore the origin of the resistance with the E10 model*.

The sensitivities were computed using the Direct Differential Method (DDM), as mentioned at the beginning of Sect. 4 and it is reported more in details in Supplement A. As evident from Fig. 4b-d, different parameters have different sensitivity on a different phase orbit with $n_{T0}$ more sensitive under treatment and $n_{R}$ or $q_{T:R}$ more sensitive out of treatment. DDM not only demonstrates the stability of the results obtained but also adds extra information on when a model is sensitive to a parameter change. This result is significant when dealing with models with varying behavior on- and off-treatment.

### Hirata et al. (2010)

**Fig. 5:**
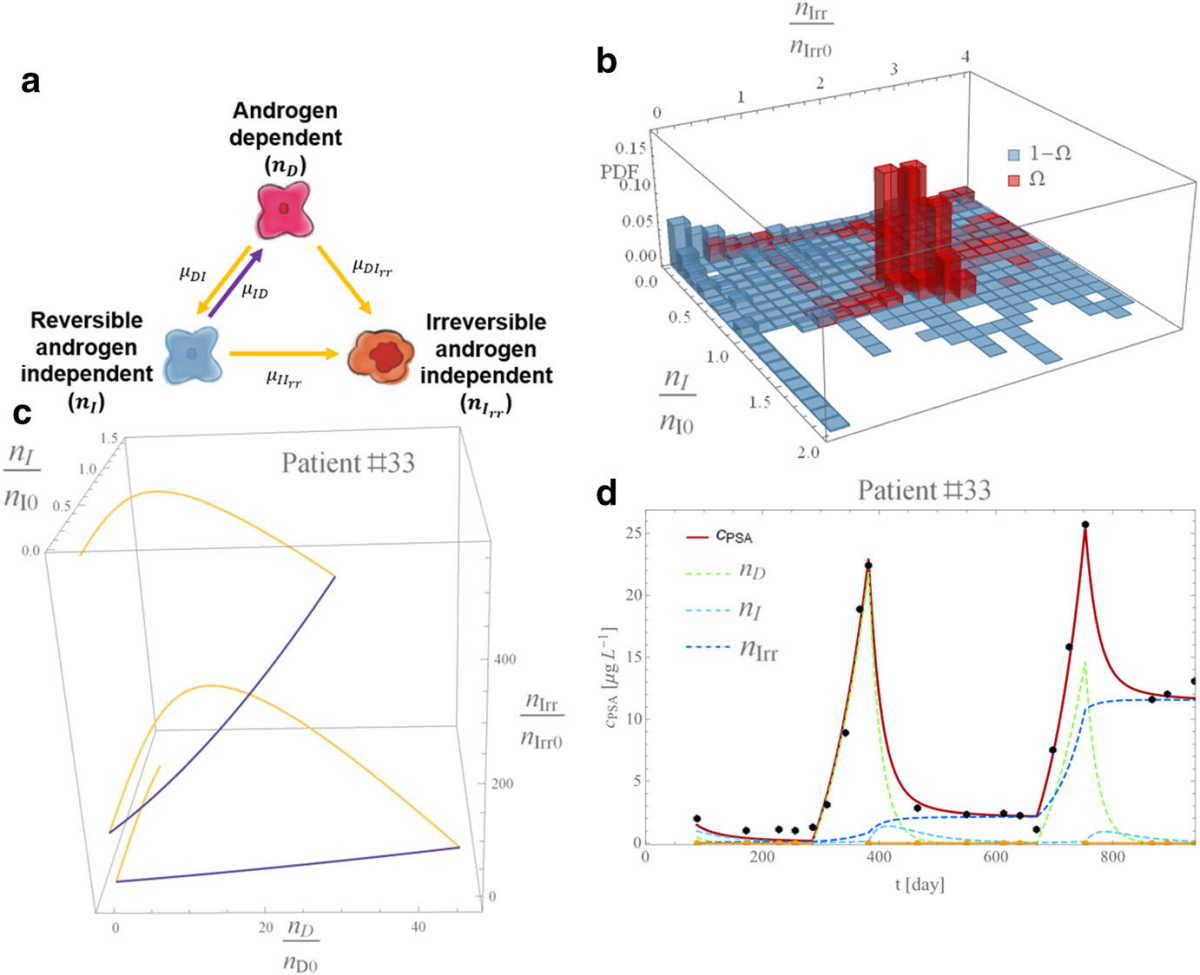
Hirata et al. model analysis results. **a**. Model schematic: under treatment (yellow arrows) and off-treatment (violet arrow). **b** PDF on the *n*_*I*_ and *n*_*D*_ space as phase space density histograms for resistant ($\Omega$, red) and responsive ($\neg \Omega$, blue) patients. **c**. Flex in the PSA profile under treatment and off-treatment in the *n*_*D*_, *n*_*I*_ and *n*_Irr_ phase space for patient #33. Colors match the sketch of panel **a**. **d**. PSA density profile (red curve), with data points with error bar (black dots). The *n*_*I*_, *n*_*D*_ and *n*_Irr_ populations are shown by the dashed blue, dashed green), and dashed cyan curves, respectively. Yellow lines along the x-axis show the intervals of treatment (Color figure online)

A series of studies (Tanaka et al. 2010; Hirata et al. 2012; Hirata and Aihara 2015) motivated the model by Hirata et al. 2010 (hereafter model H10) to capture intermittent ADT dynamics. The model is based on the coupled AD-AI population cells, supplemented with a population of irreversible AI cells, AI-Irr representing the first three-compartment model in the literature (Fig. 5a). Here we report the mathematical formulation in the proposed framework’s formalism and refer to the original paper for a detailed model description. The SoE reads with our generalized notation:

10 $$\begin{array}{*{20}l} {\frac{{{\text{d}}n_{D} }}{{{\text{d}}t}} = n_{D} \left( {T_{ps} \left( {\gamma_{D}^{{{\text{on}}}} - \gamma_{D}^{{{\text{off}}}} } \right) + \gamma_{D}^{{{\text{off}}}} } \right) + \mu_{\textit{ID}} \left( {1 - T_{ps} } \right)n_{I} ,} \hfill \\ {\frac{{{\text{d}}n_{I} }}{{{\text{d}}t}} = \mu_{{\textit{DI}}} T_{ps} n_{D} + n_{I} \left( {T_{ps} \left( {\gamma_{I}^{{{\text{on}}}} - \gamma_{I}^{{{\text{off}}}} } \right) + \gamma_{I}^{{{\text{off}}}} } \right),} \hfill \\ {\frac{{{\text{d}}n_{Irr} }}{{{\text{d}}t}} = \mu_{DIrr} T_{ps} n_{D} + \mu_{IIrr} T_{ps} n_{I} + n_{Irr} \left( {T_{ps} \left( {\gamma_{Irr}^{{{\text{on}}}} - \gamma_{Irr}^{{{\text{off}}}} } \right) + \gamma_{Irr}^{{{\text{off}}}} } \right),} \hfill \\ \end{array}$$

$n_{D} \left( {t_{0D} } \right) = n_{D0} ,{ }n_{I} \left( {t_{0I} } \right) = n_{I0} , n_{Irr} \left( {t_{0Irr} } \right) = n_{Irr0} ,$ where terms retain the identical biological meaning as previously described and the two irreversible, and reversible changes in the AI cell population are considered with the relative growth rate $\gamma_{i}^{{{\text{on}}/{\text{off}}}}$ on- and off-treatment with $i \in \left\{ {D,I,Irr} \right\}$. The serum concentration is computed as in Eq. (5) for $i \in \left\{ {D,I,Irr} \right\}$.

#### The Model in the Context of the Data

During both on- and off-treatment cycles, nullclines analysis leads to $\left\{ {n_{D} ,n_{I} ,n_{{{\text{Irr}}}} } \right\}_{{{\text{eq}}}} = \left\{ {0,0,0} \right\}$ as the only equilibrium point. By setting $a \equiv \gamma_{D}^{{{\text{off}}}} + \gamma_{I}^{{{\text{off}}}}$ $b \equiv \gamma_{D}^{{{\text{on}}}} + \gamma_{I}^{{{\text{on}}}}$, $c \equiv \gamma_{D}^{{{\text{off}}}} - \gamma_{I}^{{{\text{off}}}}$ and $d \equiv \gamma_{D}^{{{\text{on}}}} - \gamma_{I}^{{{\text{on}}}} ,$ with the discriminant ${\Delta }$ implicitly defined by the relation ${\Delta }^{2} = c^{2} + T_{ps} \left( {T_{ps} \left( {(c - d)^{2} - 4\mu_{{{\text{{\textit{DI}}}}}} \mu_{{{\text{\textit{ID}}}}} } \right) - 2c\left( {c - d} \right) + 4\mu_{{{\text{{\textit{DI}}}}}} \mu_{{{\text{\textit{ID}}}}} } \right)$, we can write the generalized eigenvalues of the Jacobian at the equilibrium as $\lambda_{1} = \gamma_{Irr}^{{{\text{off}}}} + T_{ps} \left( {\gamma_{Irr}^{{{\text{on}}}} - \gamma_{Irr}^{{{\text{off}}}} } \right)$ and the other two $\lambda_{2,3}$ in a compact form as $\lambda_{2,3} = \frac{1}{2}\left( {T_{ps} \left( {b - a} \right) + a \pm {\Delta }} \right)$. This result implies that the equilibrium is stable on-treatment and unstable off-treatment.

The phase space shows that responsive and resistant patients cluster differently on the phase-state variables. Figure 5b shows that the probability density function for the best-fit patient groups around the initial value for $n_{I} \cong n_{I0}$ and $n_{Irr} \cong 2.1n_{Irr0}$. Thus, the irreversible component of the model offers a potential tool to disentangle patient responses from the model fitting. As the resistant patients are expected to increase their irreversible cell component (i.e., asymptotically $n_{Irr} \succ n_{Irr0}$ with “$\succ$” meaning asymptotic greater), we note that $n_{I} \ll n_{I0}$ in responsive patients.

The model structure allows for the simulation of various PSA profiles thanks to the introduction of a new degree of freedom carried out with the third-compartment equations. Figure 5c shows the phase space plane for an example taken from the ${\Omega }$ set of patients (Patient #33), while Fig. 5d shows the quality of the captured PSA concentration $c_{PSA}$ profile achieved by this model.

### Portz et al. (2012)

**Fig. 6:**
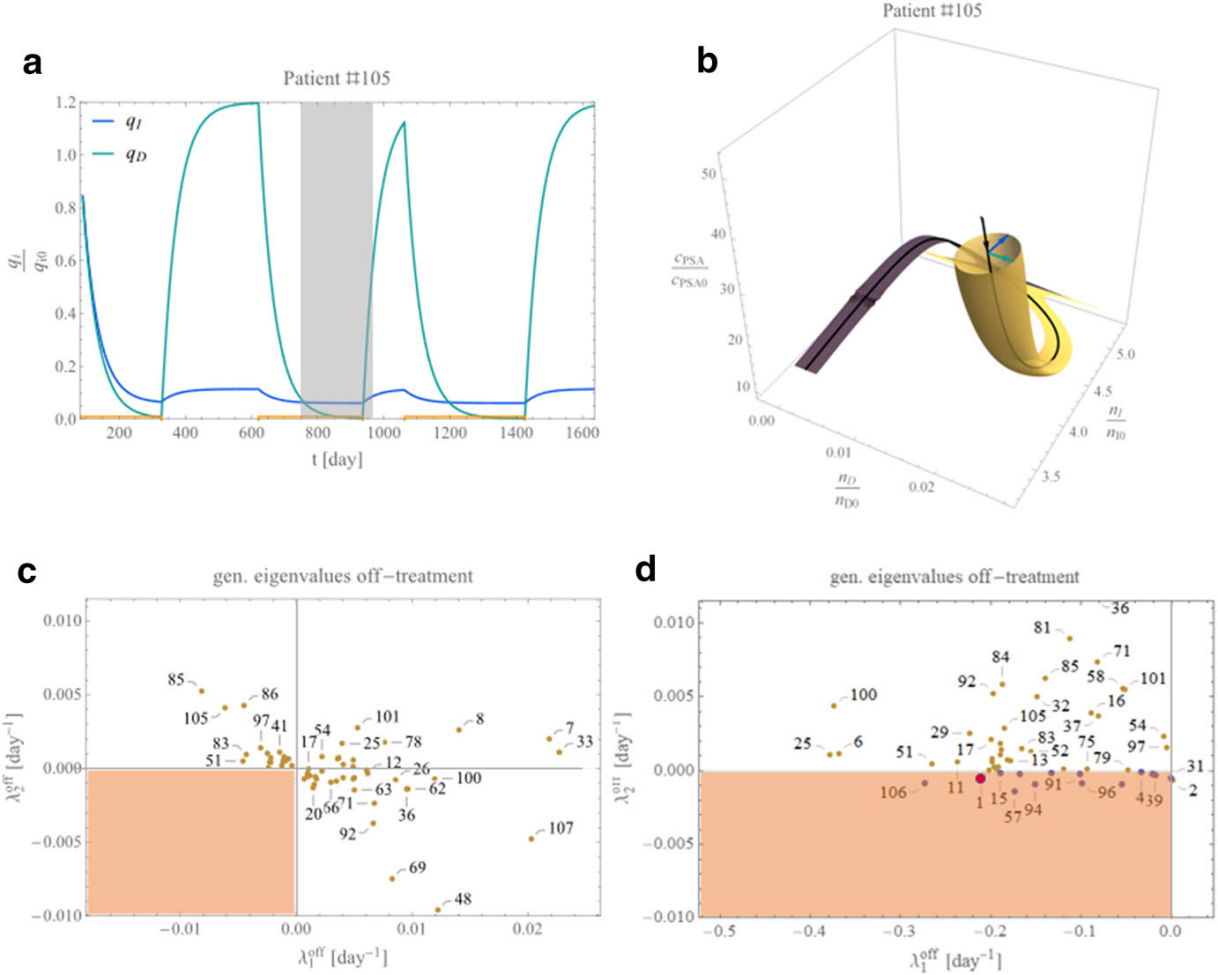
Portz et al. model analysis results. **a** Quotas for dependent and independent cells shaping the tube plot of panel **b** for a particular patient. The yellow dot–lines represent the on-treatment periods, and blue and green are the model’s independent and dependent quota levels, respectively. **b** The quota profiles are represented as a cross section. The orbital evolution corresponds to the gray box time interval in panel **a**. The blue and green arrows represent the independent and dependent quota levels of the model, respectively. On the orbit section, the tube cross section has been computed considering *q*_*D*_ along the normal *N* and *q*_*I*_ along the binormal *B* and by solving the Frenet–Serret formulas (assuming the vector field of the system under consideration being along the tangent *T*). **c**, **d** The distribution of the eigenvalues $\lambda_{1}^{{{\text{off}}}}$ and $\lambda_{2}^{{{\text{off}}}}$ off-treatment for models P12B and P12A, respectively (Color figure online)

The Portz et al. (2012) model is based on the cell quota concept (Droop 1968), which is modeled as:

11 $$\frac{{{\text{d}}q_{i} }}{{{\text{d}}t}} = \frac{{v_{{{\text{max}}}} \left( {q_{{{\text{max}}}} - q_{i} } \right)\left( {1 - T_{ps} } \right)}}{{\left( {q_{{{\text{max}}}} - q_{{i{\text{min}}}} } \right)\left( {k_{q/2} - T_{ps} + 1} \right)}} - \delta_{q} q_{i} + \gamma_{{{\text{max}}}} \left( {q_{{i{\text{min}}}} - q_{D} } \right),$$

with $q\left( {t_{0i} } \right) = q_{0i}$ for $i \in \left\{ {D,I} \right\}.$ The cell quota can grow to the maximum cell quota rate $\gamma_{{{\text{max}}}}$ and degrades at a constant rate $\delta_{q}$, with $q_{{{\text{max}}}}$ representing the shared max cell quota, $v_{{{\text{max}}}}$ the maximum cell quota uptake rate, $q_{{i{\text{min}}}} < q_{{{\text{max}}}}$ the minimum cell quota for androgen, and $1 \ne k_{q/2} > 0$ the uptake rate half-saturation level (Packer et al. 2011). The authors allow mutation between both cell populations, from AD to AI and vice versa, at rates $\mu_{{\textit{DI}}}$ and $\mu_{\textit{ID}}$ given, respectively, by the Hill’s equations of index $m = 2$:

12 $$\begin{array}{*{20}l} {\mu_{{\textit{DI}}} \left( q \right) = \mu_{{\textit{DI}}\text{max} } \frac{{k_{{\textit{DI}}/2}^{m} }}{{q^{m} + k_{{\textit{DI}}/2}^{m} }},} \hfill & {\mu_{\textit{ID}} \left( q \right) = \mu_{{\textit{ID}}{\text{max}}} \frac{{q^{m} }}{{q^{m} + k_{ID/2}^{m} }},} \hfill \\ \end{array}$$

where $\mu_{{{\textit{DI}}{\text{max}}}}$ is the maximum AD to AI mutation rate, $\mu_{{{\textit{ID}}{\text{max}}}}$ is the maximum AI to AD mutation rate, and $k_{{\textit{DI}}/2}^{m}$ and $k_{{\textit{ID}}/2}^{m}$ are the cells mutation rate half-saturation level. The model follows the evolution of AD/AI cell populations, $n_{D}$ and $n_{I}$, respectively, with the following equations:

13 $$\begin{array}{*{20}l} {\frac{{{\text{d}}n_{D} }}{{{\text{d}}t}} = n_{D} \left( { - \delta_{D} - \mu_{{\textit{DI}}\text{max} } \frac{{k_{{\textit{DI}}/2}^{2} }}{{k_{{\textit{DI}}/2}^{2} + q_{D}^{2} }} + \gamma_{\text{max} } \left( {1 - \frac{{q_{D\text{min} } }}{{q_{D} }}} \right)} \right) + \mu_{{\textit{ID}}{\text{max}}} n_{I} \frac{{q_{I}^{2} }}{{k_{ID/2}^{2} + q_{I}^{2} }},} \hfill \\ {\frac{{{\text{d}}n_{I} }}{{{\text{d}}t}} = n_{I} \left( { - \delta_{I} - \mu_{{\textit{ID}}{\text{max}}} \frac{{q_{I}^{2} }}{{k_{ID/2}^{2} + q_{I}^{2} }} + \gamma_{\text{max} } \left( {1 - \frac{{q_{I\text{min} } }}{{q_{I} }}} \right)} \right) + \mu_{{\textit{DI}}\text{max} } n_{D} \frac{{k_{{\textit{DI}}/2}^{2} }}{{k_{{\textit{DI}}/2}^{2} + q_{D}^{2} }},} \hfill \\ \end{array}$$

for $q_{i} \left( t \right) \ne 0\forall t$ and i.c. $n_{D} \left( {t_{0D} } \right) = n_{D0}$ and $n_{I} \left( {t_{0I} } \right) = n_{I0}$. The cell apoptosis and proliferation rates are, respectively, given by $\delta_{i}$ and $\gamma_{i}$ for $i = \left\{ {D,I} \right\}$. The authors model the quota for both AD and AI cell populations independently. In general, we assume $q_{{I{\text{min}}}} < q_{{D{\text{min}}}}$ to ensure that AI cells have a greater proliferation capacity in low androgen environments and $n_{D} \left( {t_{0D} } \right) \cong 0$ with $t_{0D}$ soon after treatment, as well as $n_{I} \left( {t_{0I} } \right) \cong 0$ at $t_{0I}$ at the beginning of the first treatment. Furthermore, a communal maximum proliferation rate $\gamma_{{{\text{max}}}}$ between the two populations is assumed. Both AD and AI cells produce PSA at a baseline rate $\gamma_{{{\text{PSA}}0}}$ under the androgen dependence specified by:

14 $$\frac{{{\text{d}}c_{PSA} }}{{{\text{d}}t}} = n_{D} \left( {\gamma_{{{\text{PSA}}0}} + \frac{{\gamma_{{{\text{PSA}},D}} q_{D}^{2} }}{{k_{{{\text{PSA}},D/2}}^{2} + q_{D}^{2} }}} \right) - c_{{{\text{PSA}}}} \delta_{{{\text{PSA}}}} + n_{I} \left( {\gamma_{{{\text{PSA}}0}} + \frac{{\gamma_{{{\text{PSA}},I}} q_{I}^{2} }}{{k_{{{\text{PSA}},I/2}}^{2} + q_{I}^{2} }}} \right),$$

with $c_{PSA} \left( {t_{0PSA} } \right) = c_{PSA0}$, and where $k_{PSA,i/2}$ are the half-saturation rates and $\gamma_{PSA,i}$ the growth rates, for $i = \left\{ {D,I} \right\}$. Several variants of this quota model can be found in the literature. In the present work, we consider only a couple of them. A detailed comparison between (Hirata et al. 2010) and (Portz et al. 2012) can be found elsewhere (Everett et al. 2014). The model’s complexity is demonstrated with a tube plot (Fig. 6a, b).

#### P12A in the Context of the Data

The model is an extension of the models by Ideta et al., shown in Sect. 4.1, where the equation of the quota decouples from the two cell populations behavior. Nevertheless, the quota evolution $q = q\left( t \right)$, common to $n_{D}$ and $n_{I}$, is generally smoother than $c_{A} = c_{A} \left( t \right)$ in I08A or I08B hence not justifying the approximations worked out in those models. In P12A, the only equilibrium point is at $\left\{ {n_{D} ,n_{I} } \right\}_{{{\text{eq}}}}^{{{\text{on}}/{\text{off}}}} = \left\{ {0,0} \right\}$ and for the decoupled quota equation at $q_{{{\text{eq}}}}^{{{\text{on}}}} = \frac{{\gamma_{{{\text{Dmax}}}} q_{{{\text{min}}}} }}{{\gamma_{{{\text{Dmax}}}} + \delta_{q} }}$ and $q_{{{\text{eq}}}}^{{{\text{off}}}} = \frac{{\gamma_{{D{\text{max}}}} \left( {k_{q/2} + 1} \right)q_{{{\text{min}}}} \left( {q_{{{\text{max}}}} - q_{{{\text{min}}}} } \right) + q_{{{\text{max}}}} v_{{{\text{max}}}} }}{{\left( {k_{q/2} + 1} \right)\left( {\gamma_{{D{\text{max}}}} + \delta_{q} } \right)\left( {q_{{{\text{max}}}} - q_{{{\text{min}}}} } \right) + v_{{{\text{max}}}} }}$ for the on/off-treatments, respectively. The eigenvalues at this equilibrium point are real and negative along the direction of $n_{D}$ and $n_{I}$: $\lambda_{i} \in {\mathbb{R}}_{0}^{ - }$, for $i = 1,2$ both on- and off-treatment. In the decoupled $q$ direction, the generalized eigenvalues $\lambda_{3}^{{{\text{on}}}} = - \left( {\gamma_{{D{\text{max}}}} + \delta_{q} } \right)$ and $\lambda_{3}^{{{\text{off}}}} = \lambda_{3}^{{{\text{on}}}} - \frac{{v_{{{\text{max}}}} }}{{\left( {k_{q/2} + 1} \right)\left( {q_{{{\text{max}}}} - q_{{{\text{min}}}} } \right)}}$ are always negative, leading to a node (attractor).

Nevertheless, we note that from the plot in Fig. 6c, how the best-fit solutions obtained from our inference work for all patients with this model falls in the area where $\lambda_{i} > 0$, for both $i = 1$ and $i = 2$, i.e., we are never in the presence of an attractor (off-treatment). Therefore, patient dynamics never intercept an area of the parameters’ space defined by the hyperplane that would (eventually asymptotically) lead to the annihilation of the $n_{D}$ and $n_{I}$ cell population, i.e., a steady state or a reduction of the disease present under the detection threshold. This plot is compared with the companion model in the next section, which simplifies P12A.

#### P12B in the Context of the Data

In this model, the authors extend the use of the quota concept to both $n_{D}$ and $n_{I}$ individually, i.e., fully exploiting Eq. (11), but retaining the same proliferation rate $\gamma_{{{\text{max}}}}$. The large number of parameters required by the model makes the posterior maximization time-consuming and computationally expensive in the Bayesian framework, especially in a fully numerical nested sample approach (Skilling 2004) or using differential evolution optimization tool (Feoktistov 2006; Goode and Annin 2015). For this reason, a first inference approach has been performed within Laplace approximation and followed up at the patient-specific level where judged necessary.^5^

As in P12A, the P12B critical points are $\left\{ {n_{D} ,n_{I} ,c_{PSA} } \right\}_{{{\text{eq}}}} = \left\{ {0,0,0} \right\}$ both on- and off-treatment, while for the decoupled quota equation-stability points are found at $q_{{i,{\text{eq}}}}^{{{\text{on}}}} = \frac{{\gamma_{{{\text{max}}}} }}{{\delta_{q} + \gamma_{{{\text{max}}}} }}q_{{i{\text{min}}}}$ and $q_{{i,{\text{eq}}}}^{{{\text{off}}}} = a^{ - 1} \mu_{{{\text{max}}}} q_{{i{\text{min}}}} \left( {k_{q/2} + 1} \right)\left( {q_{{{\text{max}}}} - q_{{i{\text{min}}}} } \right) + q_{{{\text{max}}}} v_{{{\text{max}}}}$ with $a \equiv v_{{{\text{max}}}} - \left( {k_{q/2} + 1} \right)\left( {\delta_{q} + \gamma_{{{\text{max}}}} } \right)\left( {q_{{i{\text{min}}}} - q_{{{\text{max}}}} } \right) \ne 0$, $\delta_{q} \ne 0$ and $\gamma_{{{\text{max}}}} \ne 0$ and for $i \in I,D$. As in P12A, the three generalized eigenvalues of the Jacobian at the equilibrium are always negative. Equations for the generalized eigenvalues $\lambda_{i}^{{{\text{on}}/{\text{off}}}}$ for $i = 1,2$ along the quota directions are analytically available but slightly cumbersome; vice versa, more interesting is the plot of $\lambda_{i}^{{{\text{off}}}}$ for $i = 1,2$ shown in Fig. 6d. The P12B solutions distribute a small number of patients in the P12A inaccessible area of double negative generalized eigenvalues (orange square in Fig. 6c, d). *In this zone of the P12B parameter space off-treatment, the model predicts a constrained (or asymptotically constrainable) tumor cell population.* Finally, we note how P12A is nested in P12B. Thus, P12B always obtains a better score in the same data representation but suffers from overfitting. We investigate this problem and offer a solution in Sect. 5 in the context of the Bayesian model comparison.

### Morken et al. (2014)

In Morken et al. (2014), the authors extend model P12B by adding ADT-induced apoptosis of prostate cancer cells in addition to the inhibition of their growth and proliferation. Therefore, the model (hereafter M14) implements the per capita mortality of androgen-dependent and independent populations introduced in the previous section with the equation:

15 $$\delta_{i} \left( {q_{i} } \right) = \delta_{i\max } \frac{{k_{i/2}^{2} }}{{q_{i}^{2} + k_{i/2}^{2} }},$$

where $k_{i/2}$ for $i \in \left\{ {D,I} \right\}$ are the apoptosis and half-saturation levels for the dependent and independent populations, respectively. We consider the SoE in the form of:

16 $$\begin{gathered} \frac{{{\text{d}}n_{D} }}{{{\text{d}}t}} = n_{D} \left( { - \delta_{D} - \frac{{\delta_{D\text{max} } k_{D\delta /2}^{2} }}{{k_{D\delta /2}^{2} + q_{D}^{2} }} - \frac{{k_{{\textit{DI}}/2}^{2} \mu_{{\textit{DI}}\text{max} } }}{{k_{{\textit{DI}}/2}^{2} + q_{D}^{2} }} + \gamma_{\text{max} } \left( {1 - \frac{{q_{D\text{min} } }}{{q_{D} }}} \right)} \right) + \frac{{\mu_{{\textit{ID}}{\text{max}}} n_{I} q_{I}^{2} }}{{k_{ID/2}^{2} + q_{I}^{2} }}, \hfill \\ \frac{{{\text{d}}n_{I} }}{{{\text{d}}t}} = \frac{{k_{{\textit{DI}}/2}^{2} \mu_{{\textit{DI}}\text{max} } n_{D} }}{{k_{{\textit{DI}}/2}^{2} + q_{D}^{2} }} + n_{I} \left( { - \delta_{I} - \frac{{\delta_{I\text{max} } k_{I\delta /2}^{2} }}{{k_{I\delta /2}^{2} + q_{I}^{2} }} - \frac{{\mu_{{\textit{ID}}{\text{max}}} q_{I}^{2} }}{{k_{ID/2}^{2} + q_{I}^{2} }} + \gamma_{\max } \left( {1 - \frac{{q_{I\text{min} } }}{{q_{I} }}} \right)} \right), \hfill \\ \end{gathered}$$

for $q_{i} \left( t \right) \ne 0{ }\forall t$ and i.c. $n_{D} \left( {t_{0D} } \right) = n_{D0}$ and $n_{I} \left( {t_{0I} } \right) = n_{I0}$, together with the equivalent of Eq. (14):

17 $$\frac{{{\text{d}}c_{{{\text{PSA}}}} }}{{{\text{d}}t}} = - c_{{{\text{PSA}}}} \delta_{{{\text{PSA}}}} + n_{D} \left( {\gamma_{{{\text{PSA}}0}} + \frac{{\gamma_{{{\text{PSA}},D}} q_{D}^{2} }}{{k_{{{\text{PSA}},D/2}}^{2} + q_{D}^{2} }}} \right) + n_{I} \left( {\gamma_{{{\text{PSA}}0}} + \frac{{\gamma_{{{\text{PSA}},I}} q_{I}^{2} }}{{k_{{{\text{PSA}},I/2}}^{2} + q_{I}^{2} }}} \right),$$

with i.c. $c_{{{\text{PSA}}}} \left( {t_{{0{\text{PSA}}}} } \right) = c_{{{\text{PSA}}0}}$. Furthermore, the same notation as in the models by Portz et al. is followed and not repeated here.

#### The Model in the Context of the Data

The analytical treatment is analogous to P12B but enriched in the dynamics variety for the extra parameters introduced in Eq. (15), although without changing equilibrium points.^6^ Our model analysis did not report other notable features.

### Baez and Kuang (2016)

The model by Baez and Kuang (2016) presents a variant of the P12A model that is able to fit PSA and androgen dynamics, thus improving PSA trend forecasting. Two models are presented in the authors’ work and considered here. The first (hereafter B16A) is a single population model of cellular concentration $n$, and two equations are coupled with it, for $\delta_{{{\text{max}}}}$ the time-dependent (over a timescale $\tau_{{\delta_{{{\text{max}}}} }}$) maximum baseline cell death rate and $c_{PSA}$ the PSA concentration that are modeled as:

18 $$\begin{array}{*{20}l} {\frac{{{\text{d}}n}}{{{\text{d}}t}} = n\left( { - n\delta - \frac{{k_{n/2} \delta_{{{\text{max}}}} }}{{q + k_{n/2} }} - \frac{{\gamma_{{{\text{max}}}} q_{{{\text{min}}}} }}{q} + \gamma_{{{\text{max}}}} } \right),} \hfill \\ {\frac{{{\text{d}}\delta_{{{\text{max}}}} }}{{{\text{d}}t}} = - \tau_{{\delta_{{{\text{max}}}} }} \delta_{{{\text{max}}}} ,} \hfill \\ {\frac{{{\text{d}}c_{{{\text{PSA}}}} }}{{{\text{d}}t}} = q\left( {\gamma_{{{\text{PSA}}1}} n + \gamma_{{{\text{PSA}}0}} } \right) - \delta_{{{\text{PSA}}}} c_{{{\text{PSA}}}} ,} \hfill \\ \end{array}$$

and a decoupled equation for androgen level:

19 $$\frac{dq}{{dt}} = \gamma \left( {q_{{{\text{max}}}} - q} \right) - \gamma_{{{\text{max}}}} \left( {q - q_{{{\text{min}}}} } \right),$$

with $n\left( {t_{0n} } \right) = n_{0} , c_{PSA} \left( {t_{0PSA} } \right) = c_{PSA0,} \delta_{{{\text{max}}}} \left( {t_{{0\delta {\text{max}}}} } \right) = \delta_{{0{\text{max}}}}$, and $q\left( {t_{0q} } \right) = q_{0} > 0$ strictly. The quota $q \ne 0\forall t$ is produced at a rate $\gamma = \gamma_{1} T_{ps} + \gamma_{2}$.

In the same work, the authors also presented a two-populations model tracking both sensitive $n_{D}$ and independent $n_{I}$ cell evolution (hereafter B16B). By implementing their SoE within the approximation that all the cells have, on average, the same mass and density, we can recast their SoE in the form:

20 $$\begin{gathered} \frac{{{\text{d}}n_{D} }}{{{\text{d}}t}} = n_{D} \left( { - \frac{{\delta_{D\text{max} } k_{D/2} }}{{q + k_{D/2} }} - \frac{{k_{{\textit{DI}}/2} \mu_{{\textit{DI}}\text{max} } }}{{q + k_{{\textit{DI}}/2} }} + \gamma_{\text{max} } \left( {1 - \frac{{q_{D\text{min} } }}{q}} \right)} \right) - \delta_{D} n_{D}^{2} , \hfill \\ \frac{{{\text{d}}n_{I} }}{{{\text{d}}t}} = \frac{{k_{{\textit{DI}}/2} \mu_{{\textit{DI}}\text{max} } n_{D} }}{{q + k_{{\textit{DI}}/2} }} + n_{I} \left( {\gamma_{\text{max} } \left( {1 - \frac{{q_{I\text{min} } }}{q}} \right) - \frac{{\delta_{I\text{max} } k_{I/2} }}{{q + k_{I/2} }}} \right) - \delta_{I} n_{I}^{2} , \hfill \\ \frac{{{\text{d}}q}}{{{\text{d}}t}} = - q\left( {\gamma_{2} + \gamma_{\text{max} } + \gamma_{1} T_{ps} } \right) + \frac{{\gamma_{\text{max} } \left( {q_{D\text{min} } n_{D} + q_{I\text{min} } n_{I} } \right)}}{{n_{D} + n_{I} }} + q_{\text{max} } \left( {\gamma_{2} + \gamma_{1} T_{ps} } \right), \hfill \\ \frac{{{\text{d}}c_{{{\text{PSA}}}} }}{{{\text{d}}t}} = q\left( {\gamma_{{{\text{PSA}}0}} + \gamma_{{{\text{PSA}}1}} \left( {n_{D} + n_{I} } \right)} \right) - \delta_{{{\text{PSA}}}} c_{{{\text{PSA}}}} , \hfill \\ \end{gathered}$$

for $n_{i} , i \in \left\{ {D,I} \right\}$ never contemporaneously null, with initial conditions $n_{D} \left( {t_{0D} } \right) = n_{D0}$, $n_{I} \left( {t_{0I} } \right) = n_{I0}$, $q\left( {t_{0q} } \right) = q_{0}$, and $c_{{{\text{PSA}}}} \left( {t_{{0{\text{PSA}}}} } \right) = c_{{{\text{PSA}}0}}$. The maximum AD to AI mutation rate is given by $\mu_{DImax}$. Furthermore, because AI cells, $n_{I}$, proliferate at lower androgen level it is assumed that $q_{I\text{min} } < q_{D\text{min} }$, and $\delta_{D\text{max} } > \delta_{I\text{max} }$ because independent cells are less susceptible to apoptosis by androgen deprivation than sensitive cells.

#### B16A in the Context of the Data

The decoupled quota equation presents an equilibrium at $q_{{{\text{eq}}}} = \frac{{\gamma_{{{\text{max}}}} \left( {q_{{{\text{min}}}} - q_{{{\text{max}}}} } \right)}}{{\gamma_{{{\text{max}}}} + (\gamma_{2} + \gamma_{1} T_{ps} )}} + q_{{{\text{max}}}}$ when $\gamma_{{{\text{max}}}} + (\gamma_{2} + \gamma_{1} T_{ps} ) \ne 0,$ belonging to the positive hyper-quadrant of the phase space (i.e., it is of biological interest). The remaining set in Eq. (18) shows two equilibria at $\left\{ {n,\delta_{{{\text{max}}}} ,c_{PSA} } \right\}_{{{\text{eq}}}}^{\left( 1 \right)} = \left\{ {0,0,\frac{{\gamma_{{{\text{PSA}},0}} q_{{{\text{eq}}}} }}{{\delta_{{{\text{PSA}}}} }}} \right\}$, which are always in the positive quadrant of the phase space of interest and $\left\{ {n,\delta_{{{\text{max}}}} ,c_{PSA} } \right\}_{{{\text{eq}}}}^{\left( 2 \right)} = \left\{ {\frac{{\gamma_{{{\text{max}}}} \left( {q_{{{\text{eq}}}} - q_{{{\text{min}}}} } \right)}}{{q_{{{\text{eq}}}} \delta }},0,\frac{{\delta \gamma_{{{\text{PSA}},0}} q_{{{\text{eq}}}} + \gamma_{{{\text{max}}}} \gamma_{{{\text{PSA}},1}} \left( {q_{{{\text{eq}}}} - q_{{{\text{min}}}} } \right)}}{{\delta_{{{\text{PSA}}}} \delta }}} \right\}$ with $q_{{{\text{eq}}}} \ne 0$, $\delta_{{{\text{PSA}}}} \ne 0$ and $\delta \ne 0$, which is also biologically meaningful. By studying the generalized eigenvalues, we see that the first of the equilibrium presents three negative generalized eigenvalues, one of which is always positive (i.e., it is a saddle point); the second equilibrium point produces the eigenvalues $\lambda_{1}^{\left( 2 \right)} = \gamma_{{{\text{max}}}} \left( {\frac{{q_{{{\text{min}}}} }}{{q_{{{\text{eq}}}} }} - 1} \right)$, $\lambda_{2}^{\left( 2 \right)} = - \delta_{{{\text{PSA}}}}$, $\lambda_{3}^{\left( 2 \right)} = - \tau_{{\delta_{{{\text{max}}}} }}$ and $\lambda_{4}^{\left( 2 \right)} = - \gamma_{{{\text{max}}}} - \left( {\gamma_{2} + \gamma_{1} T_{ps} } \right)$ which are all always negative, thus representing a stable point of attraction.

Due to the stability of the second equilibrium (on- and off-treatment), it is worth investigating the proximity of the patients’ orbits to the equilibria on the Poincare sections involving the PSA concentration $c_{{{\text{PSA}}}}$ we obtained from Eq. (18). Nevertheless, the low quality of the likelihood, $L\left( \bf{p} \right) = \Pr \left( {D|\bf{p}},{I} \right)$, see Sect. 3) in the ${\Omega }$ set of patients, demotivates further analysis. A single population $n$ seems to not adequately capture disease progression, which remain the primary focus of our work, making the model less attractive for clinical implications and therefore not pushed forward here.

#### B16B in the Context of the Data

The model presents cubic dependence on $q$ and quadratic on $n_{D}$.^7^ We select to investigate only the null-equilibrium point of independent and dependent cells. It is evident that $n_{D} = 0$ is an equilibrium for the first of Eq. (20). Therefore, by *assuming* $n_{D} = 0$ (and $n_{I} > 0$ strictly), we can confirm the existence of two equilibria, the first located at $\left\{ {n_{I} ,q,c_{{{\text{PSA}}}} } \right\}_{{{\text{eq}}}}^{\left( 1 \right)} = \left\{ {0,q_{{{\text{max}}}} + \frac{{\gamma_{{{\text{max}}}} \left( {q_{{I{\text{min}}}} - q_{{{\text{max}}}} } \right)}}{{\gamma_{{{\text{max}}}} + (\gamma_{2} + \gamma_{1} T_{{{\text{ps}}}} )}},\frac{{\gamma_{{{\text{PSA}}0}} }}{{\delta_{{{\text{PSA}}}} }}q_{{{\text{eq}}}} } \right\}$, for $\gamma_{{{\text{max}}}} + (\gamma_{2} + \gamma_{1} T_{{{\text{ps}}}} ) \ne 0$ and $\delta_{{{\text{PSA}}}} \ne 0$, which is of biological interest. The second, algebraically more cumbersome, reduces its nonnegativity condition to the simple one $\delta_{I\text{max} } + \frac{{\gamma \gamma_{{{\text{max}}}} \left( {q_{{I{\text{min}}}} - q_{{{\text{max}}}} } \right)}}{{\gamma_{{{\text{max}}}} q_{{I{\text{min}}}} + \gamma q_{{{\text{max}}}} }} + \frac{{\gamma \gamma_{{{\text{max}}}} \left( {q_{{I{\text{min}}}} - q_{{{\text{max}}}} } \right)}}{{k_{I/2} \left( {\gamma + \gamma_{{{\text{max}}}} } \right)}} \le 0$, that is verified over all studied patients.

Again, as explored in previous models, we are interested in the existence of negative generalized eigenvalues of the Jacobian at the equilibria off-treatment, i.e., a point of equilibrium with an asymptotic constrained expansion of the tumoral cell population. Despite the model complexity, it is easy to prove numerically that the Jacobian for both equilibrium points has at least one positive generalized eigenvalue, making these points saddle points that are not of interest to us.

### Elishmereni et al. (2016)

The Elishmereni et al. (Elishmereni et al. 2016) model accounts for two dynamics: disease dynamics represented by PSA used as a proxy for tumor volume and the pharmacology dynamics combined with the emergence of resistant cells from androgen receptor-independent $n_{I}$ and testosterone androgen receptor-dependent $n_{IAR}$ mechanism. The PSA concentration $c_{PSA}$ of interest to us, is governed by the following numerically highly complex SoE^8^:

21 $$\begin{aligned} & \frac{{{\text{d}}c_{{{\text{PSA}}}} }}{{{\text{d}}t}} = \widehat{{\underline {c} }}_{{{\text{PSA}}}} \gamma_{{t{\text{PSA}}}} {\text{min}}\left( {\gamma_{{{\text{PSA}}}} c_{{{\text{PSA}}}}^{K} ,\frac{{{\text{log}}2}}{{\gamma_{{{\text{PSAmax}}}} }}} \right) \\  & \qquad+ \eta_{{T,{\text{PSA}}}} \left( {c_{{{\text{PSA}}}} - \frac{{\tilde{c}_{{{\text{PSA}}}} }}{2}} \right)^{ + } \left( {\eta_{I,T} R_{I} \widehat{{\underline {c} }}_{PSA} + n_{T} - 1} \right), \hfill \\ & \frac{{{\text{d}}n_{T} }}{{{\text{d}}t}} = \frac{{\gamma_{T} \left( {1 - T_{ps} } \right)}}{{\eta_{H,T} H + 1}} - \gamma_{T} n_{T} , \hfill \\ & \frac{{{\text{d}}H}}{{{\text{d}}t}} = T - \frac{{\delta_{T} l_{H}^{\max } He^{{R_{T:AR} }} }}{{e^{{R_{T:AR} }} + l_{H}^{\max } }}, \hfill \\ & \frac{{{\text{d}}R_{T:AR} }}{{{\text{d}}t}} = \gamma_{T:AR} T\widehat{{\underline {R} }}_{T:AR} , \hfill \\ & \frac{{{\text{d}}R_{I} }}{{{\text{d}}t}} = \gamma_{I} T\widehat{{\underline {R} }}_{I} , \hfill \\ &\frac{{{\text{d}}K}}{{{\text{d}}t}} = - \rho_{K} , \hfill \\ & \frac{{{\text{d}}T}}{{{\text{d}}t}} = - \delta_{T} T \hfill \\ \end{aligned}$$

with $c_{{{\text{PSA}}}} \left( {t_{{0{\text{PSA}}}} } \right) = c_{{{\text{PSA}}0}}$, $n_{T} \left( {t_{{0n_{T} }} } \right) = n_{T0}$, $H\left( {t_{0H} } \right) = H_{0} ,$ $R_{T:AR} \left( {t_{0T:AR} } \right) = R_{T:AR0} , R_{I} \left( {t_{{0{\text{I}}}} } \right) = R_{I0} , K\left( {t_{0K} } \right) = K_{0}$, and $T\left( {t_{0T} } \right) = T_{0}$ with $\left( x \right)^{ + } = x\theta \left( x \right)$ ramp/positive function of the generic $x$, $\theta$ the previously introduced Heaviside step function. In the above equation $\gamma_{{{\text{PSAmax}}}}$ is the limit to the PSA growth rate, $\rho_{K}$ the K growth rate, $\eta_{{T,{\text{PSA}}}}$ the testosterone, $T$, effect on the PSA growth, $\gamma_{T}$ the instantaneous rate of change in $T$, $\eta_{H,T}$ the effect of intermediate components $H$, e.g., bound androgen receptor AR, on $T$, with same clearance rate $\delta_{T}$. $\gamma_{T:AR}$ is the increase resistance rate, $\gamma_{I}$ the increase resistance rate for testosterone-AR-independent paths $R_{I}$, and $\eta_{I,T}$ rules the effect of $R_{I}$ on the PSA growth. The growth rate of $c_{PSA}$ is given by

22 $$\gamma_{{{\text{PSA}}}} = \left\{ {\begin{array}{*{20}l} 1 \hfill & {c_{{{\text{PSA}}}} > c_{{t{\text{PSA}}}} } \hfill \\ {\sigma_{{{\text{PSA}}}} + \left( {1 - \sigma_{{{\text{PSA}}}} } \right)\frac{{c_{{{\text{PSA}}}} }}{{c_{{t{\text{PSA}}}} }}} \hfill & {c_{{{\text{PSA}}}} \le c_{{t{\text{PSA}}}} ,} \hfill \\ \end{array} } \right.$$

where $\sigma_{{{\text{PSA}}}}$ rules the steepness on the linear grown relation, $c_{{t{\text{PSA}}}}$ the PSA threshold to switch in quiescent mode. Finally, control limits $l_{i}$ $i \in \left\{ {{\text{PSA}},H,n_{I} ,n_{IAR} } \right\}$ are added by hand to handle system divergences with a “manual”-bounding scheme ($\underline{{\hat{f}}}_{i} \equiv \frac{{\left( {l_{i\max } - f_{i} } \right)^{ + } }}{{l_{i\max } }}$ for the generic function $f_{i}$).

In the practice the dynamics of the system is designed so that the instantaneous androgen rate of change $\gamma_{T}$ is saturated by a control coefficient $\eta_{{T,{\text{PSA}}}}$ through an intermediary delaying effect ruled by a delay modeling function $H$ over the ADT therapy, $T$ therapy function with scale factor $\delta_{{{\text{ADT}}}}$ and a double mechanism for androgen independence cell population depending on $\eta_{I,T}$, and not depending on $n_{I}$, the androgen receptor (with the respective scale factor $\gamma_{I}$ and $\gamma_{T:AR}$).

#### The Model in the Context of the Data

The system has no equilibria influencing its dynamics, as evident from the 6th of Eq. (21). Further analysis is done in Sect. 5 to determine how well the model performs in the Bayesian model comparison.

### Zhang et al. (2017)

Zhang et al. (2017) present a three-population competition model, based on Lotka–Volterra (LV) dynamics, where androgen-dependent $n_{D}$, androgen producing $n_{P}$, and androgen-independent cells $n_{I}$, are considered. Basing the approach on game theory, the authors derive a competition matrix $\alpha = \alpha_{ij} \;i,j \in \left\{ {D,I,P} \right\}$ based on the parametrization of growth rates $\gamma_{i}$ and carrying capacities $K_{i}$ with $i \in \left\{ {D,I,P} \right\}$ resulting in this set of algebraic-differential equations:

23 $$\begin{array}{*{20}c} {\frac{{{\text{d}}n_{D} }}{{{\text{d}}t}} = \gamma_{D} n_{D} \left( {1 - \frac{{\alpha_{11} n_{D} + \alpha_{12} n_{p} + \alpha_{13} n_{I} }}{{n_{p} \left( {\beta - T_{ps} + 1} \right)}}} \right),} \\ {\frac{{{\text{d}}n_{P} }}{{{\text{d}}t}} = \gamma_{P} n_{p} \left( {1 - \frac{{\alpha_{21} n_{D} + \alpha_{22} n_{p} + \alpha_{23} n_{I} }}{{K_{P} }}} \right),} \\ {\frac{{{\text{d}}n_{I} }}{{{\text{d}}t}} = \gamma_{i} n_{I} \left( {1 - \frac{{\alpha_{31} n_{D} + \alpha_{32} n_{p} + \alpha_{33} n_{I} }}{{K_{I} }}} \right),} \\ \end{array}$$

where ADT is modeled by the decreasing carrying capacity with $\beta < 1$ or supporting androgen-dependent cells with $\beta > 1$. The authors considered several constraints derived from the literature and researchers’ experience to shape the model parameter influence: $\alpha_{ii} = 1\forall i$, $\alpha_{31} > \alpha_{21} ,$ $\alpha_{32} > \alpha_{12} ,$ $\alpha_{13} > \alpha_{23} ,$ $\alpha_{13} > \alpha_{21} ,$ $\alpha_{32} > \alpha_{31} ,$ and $\alpha_{ij} \in \left] {0,1} \right[\forall i \ne j$. Finally, the PSA dynamics is governed by:

24 $$\frac{{{\text{d}}c_{{{\text{PSA}}}} }}{{{\text{d}}t}} = \mathop \sum \limits_{{i \in \left\{ {D,P,I} \right\}}}^{{}} n_{i} - \delta c_{{{\text{PSA}}}} ,$$

with $\delta$ the PSA clearance rate.

#### The Model in the Context of the Data

With the coupling of Eq. (24), the system presents four equilibria, but only two are of biological interest: $\left\{ {n_{D} ,n_{P} ,n_{I} ,c_{{{\text{PSA}}}} } \right\}_{{{\text{eq}}}}^{\left( 1 \right)} = \left\{ {0,k_{P} ,0,\frac{{k_{P} }}{\delta }} \right\} \in {\mathbb{R}}_{0}^{4 + }$ and $\left\{ {n_{D} ,n_{P} ,n_{I},c_{{{\text{PSA}}}} } \right\}_{{\text{eq}}}^{\left( 2 \right)} =\Bigg\{ \frac{{k_{P} \left( {\beta - \alpha_{12} - T_{ps} + 1}\right)}}{{\alpha_{21} \left( {\beta - \alpha_{12} - T_{ps} + 1}\right) + 1}}$, $\frac{{k_{P} }}{{\alpha_{21} \left( {\beta - \alpha_{12} - T_{ps} + 1} \right) + 1}},0\frac{{k_{P} \left( {\alpha - \alpha_{12} - T_{ps} + 2} \right)}}{{\delta + \delta \alpha_{21}\left( {\beta - \alpha_{12} - T_{ps} + 1} \right)}}\Bigg\}$ where these ratios exist. For the first equilibrium, the eigenvalues of the Jacobian are positive in the $n_{D}$, $n_{P}$ and *c*_PSA_ phase space and therefore of marginal interest. Vice versa, by setting $a \equiv 1 + \beta$, $b \equiv \beta - \alpha_{12} + 1$, $d \equiv \alpha_{21} \left( {\beta - \alpha_{12} + 1} \right) + 1$ and $e \equiv \beta + \alpha_{21} (\beta - \alpha_{12} + 1)^{2} - \alpha_{12} + 1$ together with the discriminant squared $\Delta^{2} = (e\gamma_{D} + \beta \gamma_{P} + \gamma_{P} )^{2} - 4ade\gamma_{D} \gamma_{P}$, we can write the four eigenvalues of the Jacobian for the second equilibrium off-treatment as: $\lambda_{1}^{{\left( 2 \right){\text{off}}}} = - \delta ,$ $\lambda_{2}^{{\left( 2 \right){\text{off}}}} = \gamma_{I} - \frac{{\gamma_{I} k_{P} \left( {b\alpha_{31} + \alpha_{32} } \right)}}{{dk_{I} }}$, $\lambda_{3}^{{\left( 2 \right){\text{off}}}} = - \frac{{a\gamma_{P} + {\Delta } + e\gamma_{D} }}{2ad}$ and $\lambda_{4}^{{\left( 2 \right){\text{off}}}} = \frac{{{\Delta } - a\gamma_{P} - e\gamma_{D} }}{2ad}$, where the ratios exist, which are always negative for the fitted parameters, hence representing a stable equilibrium and opening the possibility to achieve an equilibrium off-treatment.

### Phan et al. (2019)

The model (hereafter P19) presented by Phan et al. (Phan et al. 2019) is a variant of the work of Sect. 4.6 (Baez and Kuang 2016) in which the third population of weakly dependent cells, *n*_*wD*_, is added to investigate the influence of extra degrees of freedom added by the new population. The death term is also adapted from Eq. (16). Retaining the notation used in Sect. 4.6, we can recast the model in the following form:

25 $$\begin{aligned} \frac{{{\text{d}}n_{D} }}{{{\text{d}}t}} & = n_{D} \left( { - \frac{{\delta_{D\text{max} } k_{D/2} }}{{q + k_{D/2} }} - \frac{{2k_{{\textit{DI}}/2} \mu_{{\textit{DI}}\text{max} } }}{{q + k_{{\textit{DI}}/2} }} + \gamma_{\text{max} } \left( {1 - \frac{{q_{D\text{min} } }}{q}} \right)} \right) \\ &\quad+ \frac{{k_{{\textit{DI}}/2} \mu_{{\textit{DI}}\text{max} } n_{wD} }}{{q + k_{{\textit{DI}}/2} }} - \delta_{D} n_{D}^{2} \\ \frac{{{\text{d}}n_{wD} }}{{{\text{d}}t}} & = n_{wD} \left( { - \frac{{2k_{{\textit{DI}}/2} \mu_{{\textit{DI}}\text{max} } }}{{q + k_{{\textit{DI}}/2} }} - \frac{{\delta_{wD\text{max} } k_{wD/2} }}{{q + k_{wD/2} }} + \gamma_{\max } \left( {1 - \frac{{q_{wD\text{min} } }}{q}} \right)} \right) \\ &\quad+ \frac{{k_{{\textit{DI}}/2} \mu_{{\textit{DI}}\text{max} } n_{D} }}{{q + k_{{\textit{DI}}/2} }} - \delta_{wD} n_{wD}^{2} \\ \frac{{{\text{d}}n_{I} }}{{{\text{d}}t}} & = \frac{{k_{{\textit{DI}}/2} \mu_{{{\textit{DI}}{\text{max}}}} \left( {n_{D} + n_{wD} } \right)}}{{q + k_{{\textit{DI}}/2} }} + n_{I} \left( {\gamma_{\text{max} } \left( {1 - \frac{{q_{I{\text{min}}}}}{q}} \right) - \frac{{\delta_{I\text{max} } k_{I/2} }}{{q + k_{I/2} }}} \right) - \delta_{I} n_{I}^{2} \\ \frac{{{\text{d}}q}}{{{\text{d}}t}} & = q\left( { - \left( {\gamma_{2} + \gamma_{1} T_{ps} } \right) - \gamma_{\text{max} } } \right) + \frac{{\gamma_{\text{max} } \left( {q_{D\text{min} } n_{D} + q_{I\text{min} } n_{I} + q_{wD\text{min} } n_{wD} } \right)}}{{n_{D} + n_{I} + n_{wD} }} \\ &\quad+ q_{\max } \left( {\gamma_{2} + \gamma_{1} T_{ps} } \right) \\ \frac{{{\text{d}}c_{{{\text{PSA}}}} }}{{{\text{d}}t}} & = q\left( {\gamma_{{{\text{PSA}}0}} + \gamma_{{{\text{PSA}}1}} \left( {n_{D} + n_{I} + n_{wD} } \right)} \right) - \delta_{{{\text{PSA}}}} c_{{{\text{PSA}}}} \\ \end{aligned}$$

with initial conditions $n_{D} \left( {t_{0D} } \right) = n_{D0} ,n_{wD} \left( {t_{0wD} } \right) = n_{wD0} ,n_{I} \left( {t_{0I} } \right) = n_{I0} ,q\left( {t_{0q} } \right) = q_{0} ,c_{{{\text{PSA}}}} \left( {t_{{0{\text{PSA}}}} } \right) =c_{{{\text{PSA}}0}}$ together with the required biological inequalities $q_{{D{\text{min}}}} > q_{{wD{\text{min}}}}$ and $q_{{D{\text{min}}}} > q_{{I{\text{min}}}}$.

#### P19 in the Context of the Data

The idea of a third population is not new and already advanced with success in the model by Hirata et al. (2010). Nevertheless, the structure of the equations in Eq. (10) is very different from the Hirata et al. model in Eq. (10), with significantly more parameters not readily justifiable within the present dataset quality. Similar considerations were already worked out by Phan et al. We remark only that the complexity of the analysis, already evident in Sect. 4.6.2, is pushed further in this context, where only numerical investigation is available for equilibria and stability. The only off-treatment equilibrium accessible by the orbits is the one for $\left\{ {n_{D} ,n_{wD,} n_{I} ,q,c_{{{\text{PSA}}}} } \right\}_{{{\text{eq}}}}^{{{\text{off}}}} = \left\{ {0,{ }0,{ }0,{ }\frac{{\gamma_{{{\text{max}}}} q_{I\,\min } + \gamma_{2} q_{{{\text{max}}}} }}{{\gamma_{2} + \gamma_{{{\text{max}}}} }},{ }\frac{{\gamma_{{{\text{PSA}}0}} \left( {\gamma_{{{\text{max}}}} q_{I\,\min } + \gamma_{2} q_{{{\text{max}}}} } \right)}}{{\delta_{{{\text{PSA}}}} \left( {\gamma_{2} + \gamma_{{{\text{max}}}} } \right)}}} \right\}$ and $\delta_{{{\text{PSA}}}} \ne 0$ which is always positive with always negative eigenvalues $\lambda_{1}^{{{\text{off}}}} = - \gamma_{2} - \gamma_{{{\text{max}}}}$ and $\gamma_{2}^{{{\text{off}}}} = - \delta_{{{\text{PSA}}}}$. This is of limited biological interest as it is not compatible with the irreversibility nature of $n_{I}$, if not by surgical castration.

### Brady-Nicholls et al. (2020)

The Brady-Nicholls et al. (2020) model (hereafter B20) is based on the hypothesis that prostate cancer stem cells’ enrichment induces resistance. The model correlates stem cell proliferation with serum PSA through SoE for the prostate cancer stem cells $n_{S}$, the non-stem (differentiated) cells $n_{D}$, and for PSA serum concentration $c_{PSA}$. We report the system in the following way:

26 $$\begin{array}{*{20}l} {\frac{{{\text{d}}n_{S} }}{{{\text{d}}t}} = \frac{{p_{S} \log \left( 2 \right)n_{S}^{2} }}{{n_{D} + n_{S} }},} \hfill \\ {\frac{{{\text{d}}n_{D} }}{{{\text{d}}t}} = \log \left( 2 \right)n_{S} \left( {1 - \frac{{p_{S} n_{S} }}{{n_{D} + n_{S} }}} \right) - \delta_{D} T_{ps} n_{D} ,} \hfill \\ {\frac{{{\text{d}}c_{{{\text{PSA}}}} }}{{{\text{d}}t}} = \gamma_{{{\text{PSA}}}} n_{D} - \delta_{{{\text{PSA}}}} c_{{{\text{PSA}}}} ,} \hfill \\ \end{array}$$

with initial conditions $n_{S} \left( {t_{0S} } \right) = n_{S0}$, $n_{D} \left( {t_{0D} } \right) = n_{D0}$ and $c_{PSA} \left( {t_{0PSA} } \right) = c_{PSA0}$. It is assumed that stem cells divide at rate log(2), and the division is either symmetric yielding two stem cells (Enderling 2015) or asymmetric, where the stem cell produces one stem and one differentiated cell. The parameter that governs this effect is $p_{s}$. The PSA differentiated cell production rate and PSA clearance rate are given by $\gamma_{PSA}$ and $\delta_{PSA}$, respectively, and $T_{ps}$ is the patient-specific treatment function (see Sect. 2.1).

#### The Model in the Context of the Data

The SoE presents an infinite set of equilibrium points when off-treatment $T_{ps} \left( t \right) = 0$ in the intersection of the plane $n_{S} \left( t \right) = 0$ with the plane given by $c_{{{\text{PSA}}}} \left( t \right) = \frac{{\gamma_{{{\text{PSA}}}} n_{D} \left( t \right)}}{{\delta_{{{\text{PSA}}}} }}$ conditional to $n_{D} \ne 0$ and $\delta_{PSA} \ne 0$ and the generalized eigenvalues of the Jacobian results in a double-zero generalized eigenvalue $\lambda_{1} = 0$, $\lambda_{2} = 0$ and a third negative eigenvalue $\lambda_{3} = - \delta_{PSA}$. Standard center manifold computation (Wiggins 2003) shows slow-2D-manifold dynamics that can be integrated to prove that the equilibria are unstable, and therefore not of interest.

## Bayesian Model Comparison

Maybe the most vital point of the Bayesian framework, and the reason for its increasing popularity, is its innate model comparison ability, based on logic as an instrument for selection. We exploit this feature here using the Bayesian factor to compare the different models in their ability to simulate the data. It should be noted that this framework innate penalizes models based on the number of parameters required. This phenomenon is sometimes referred to as the Occam’s razor factor (Jefferys and Berger 1992).

Starting from the classical Bayesian theorem, the Bayes factor $\beta_{ij}$ for PSA model $M_{i}$ over the PSA model $M_{j}$ is computed as a ratio of the probabilities of the two models (the odd-ratio, $O_{ij}$)

27 $$O_{ij} = \frac{{\Pr \left( {M_{i} ,I} \right)\Pr \left( {D|M_{i} ,I} \right)}}{{\Pr \left( {M_{j} ,I} \right)\Pr \left( {D|M_{j} ,I} \right)}} = \frac{{\Pr \left( {M_{i} ,I} \right)}}{{\Pr \left( {M_{j} ,I} \right)}}\beta_{ij} ,$$

such that, because $\sum\nolimits_{i = 1}^{{N_{m} }} {\Pr } \left( {M_{i} |D,I} \right) = 1$ (with $N_{m}$ number of models to compare) if we are interested in how a model, say $M_{1}$, compares to the other models $M_{j}$, we arrive at

28 $${\text{Pr}}\left( {M_{1} |D,I} \right) = \frac{{O_{i1} }}{{\mathop \sum \nolimits_{j = 1}^{{N_{m} }} O_{j1} }}.$$

We implement Eq. (28) to compare one patient at a time in one model against all the other models individually. For example, we are going to implement the comparison between $M_{1}$, and every other $M_{2}$ as ${\text{Pr}}\left( {M_{2} |D,I} \right) = \frac{1}{{1 + O_{21}^{ - 1} }}$, and we proceed iteratively.

We first explore the Laplace approximation framework under the assumption of equally-prioritized models, i.e., assuming that no previous preference can be accorded to any of the PSA models considered. We can exploit the asymptotic approximation (Murphy 2012; Theodoridis 2015) to the global likelihood, i.e., the evidence of the $i^{th}$ model, ${\text{Pr}}\left( {D|M_{i} } \right)$, writing

29 $$\begin{aligned} \Pr \left( {D|M_{i} } \right) & = \mathop \smallint \limits_{{}}^{{}} {\text{d}}{\bf{p}}\Pr \left({\bf{p}}|M,I \right)L\left({\bf{p}}|I \right) \\ & \cong \Pr \left( {\hat{\bf{p}}|M_{i} } \right)L\left( {\hat{\bf{p}}} \right)\sqrt {\det \left( {F\left( \bf{p} \right)} \right)} , \\ \end{aligned}$$

with $F$ being the information matrix introduced in Sect. 3.1. A classical result of Bayesian analysis is to consider the limit of the previous expression, but for an increased number of data points ($N_{p} \to \infty$) and flat priors, i.e., to compute the popular BIC index against AIC (Akaike 1974; Schwarz 1978). As the number of patient data points is often limited $N_{p} \ll \infty$ and we make explicit use of priors, BIC or AIC indices are not justifiable for model comparison. Instead, we build up a model-of-model function (Pasetto et al. 2021) to encode prior information as soon as available. Furthermore, as introduced above, we verified the Laplace approximation with fully numerical integration based on nested sampling algorithms (Skilling 2004; Mukherjee et al. 2006; Feroz and Hobson 2008), i.e., a numerical technique designed explicitly to compute the global likelihood of models with different degrees of freedom.

### Single Patient Comparison Results

**Fig. 7:**
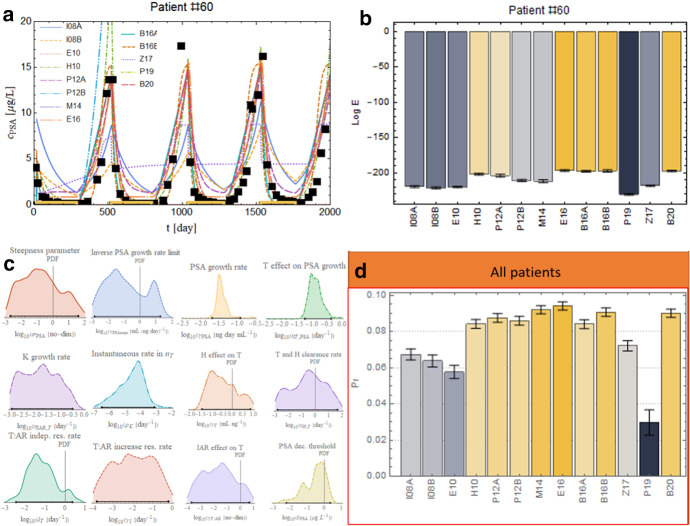
Bayesian model comparison results. **a** Best fits for the 13 models analyzed for a representative patient. The black squares are the error bars enhanced with a more prominent marker for visibility reasons; yellow lines along the x-axis represent the on-treatment periods. **b** Model log-evidence comparison on patient #60 with error bars as obtained by nest-sampling technique. The color range shows the best-performing model (yellow) and fades to the worst-performing model (gray). **c** Unnormalized posterior PDF for the best performing model, E16, and credible interval as black segment over the x-axis. **d** Comparison between the normalized log-evidence overall patient data. (The color scheme is consistent with panel b) (Color figure online)

Figure 7a shows an example of the quality of the model calibration achieved by Bayesian posterior inference introduced in Sect. 3 applied to the parameter inference problem to all the models. The simulated disease dynamics vary significantly between the different models, and discrepancies between different models and patient data may indicate likely or unlikely biological mechanisms driving individual patients’ resistance.

Model evidence (Fig. 7b) demonstrates that no single model represents all patient data accurately, suggesting that different biology drive individual patients’ responses or that no model correctly faces the PSA problem. It may also imply that the PSA dynamics alone may be insufficient to discriminate between the different biological models. For some patients, model selection identifies models with a higher probability than others, but selection varies on a per-patient basis. As a classical proof-of-concept of the Bayesian technology employed, we report for the best performing model, E16, for patient #60 the unnormalized posterior marginalized PDF for each parameter in Fig. 7c. The PDFs are almost unimodal (but not for all parameters, see next Sect. 6), suggesting that this model represents fairly the patient and that the Laplace approximation could be justified. The credible intervals for the log parameters are also plotted and superimposed to the x-axis.

### Overall Model Selection

We calculate the Bayesian maximum a posteriori performance for all the patients for each model (Fig. 7d), resulting in the Elishmereni et al. (2016) model marginally performing better on most patients. This result does not surprise us, as it is a model designed on clinical necessities, i.e., it was crafted with careful handling of the medical treatment. Nevertheless, as mentioned before, in the case of model comparison on a patient-to-patient basis, we could not identify a model that performed statistically better than the others thus eventually indicating the correct biological mechanics governing PSA dynamics. Figure 7d shows that E16 is preferred only on 10% of the patients, and eight of the 13 models have scores above the 8%.

## Conclusion and Discussion

This work considers several mathematical models (Table 1) to simulate PSA dynamics of prostate cancer response to IADT in a prospective clinical trial. We exploit Bayesian continuous and discrete inference to interpret the data and identify the model with the highest likelihood of simulating the clinically observed dynamics. Using the PSA biomarker and the comparison between the different models, we1) identify several models that can separate responding patients and patients that develop resistance to intermittent ADT through the model fitting, 2) performed the Bayesian model comparison and demonstrated that the model by Elishmereni et al. (2016) performed slightly better than the others, i.e., as a better representative of most patients in the trial. Nevertheless, as evidenced in the example of Fig. 7c, the marginalized posterior PDF is often not all optimally single-peaked, casting shadows in an attempt to use this model to solve forecast problems. While we have focused on the models’ inference to evaluate the possible connection with their underpinned biology, we will explore the potentiality and limitation of the models’ forecasting ability to predict clinical PSA trends in a follow-up paper (Pasetto et al. 2021, in preparation).

**Table 1:**
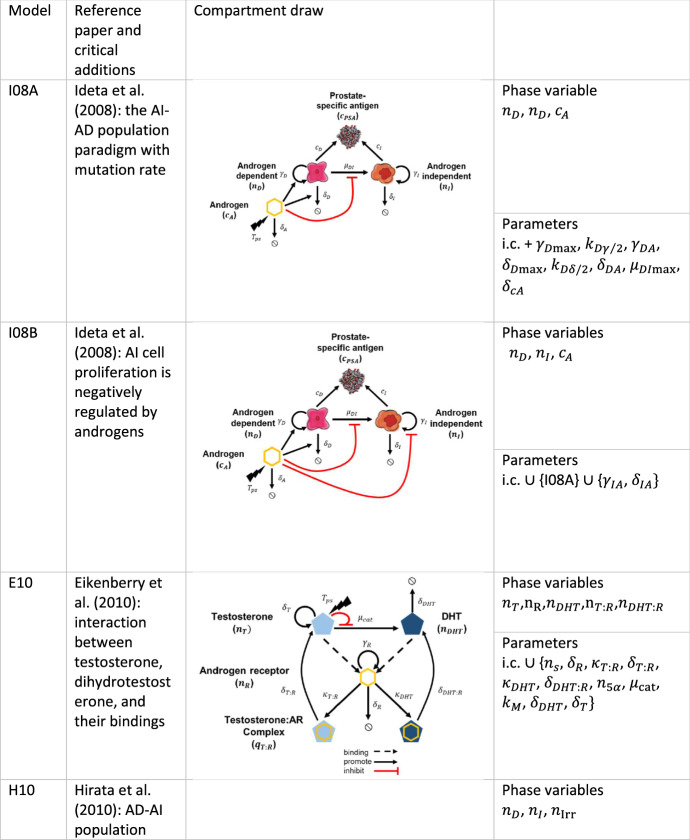

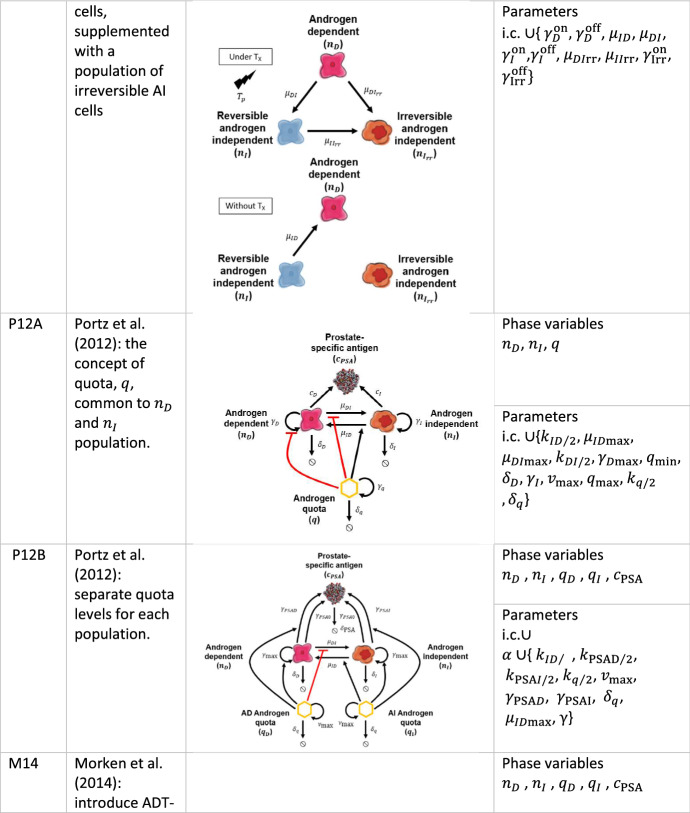

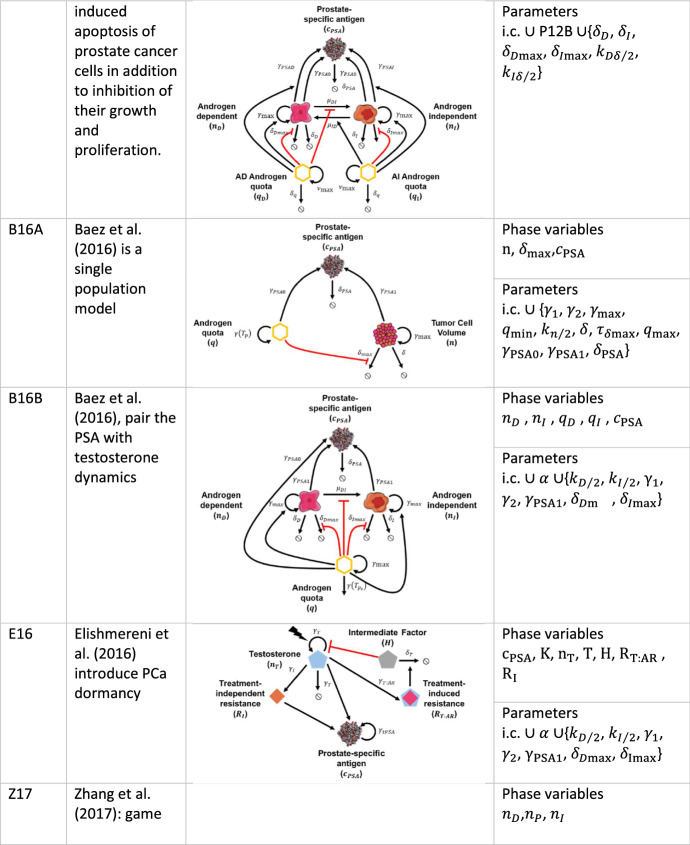

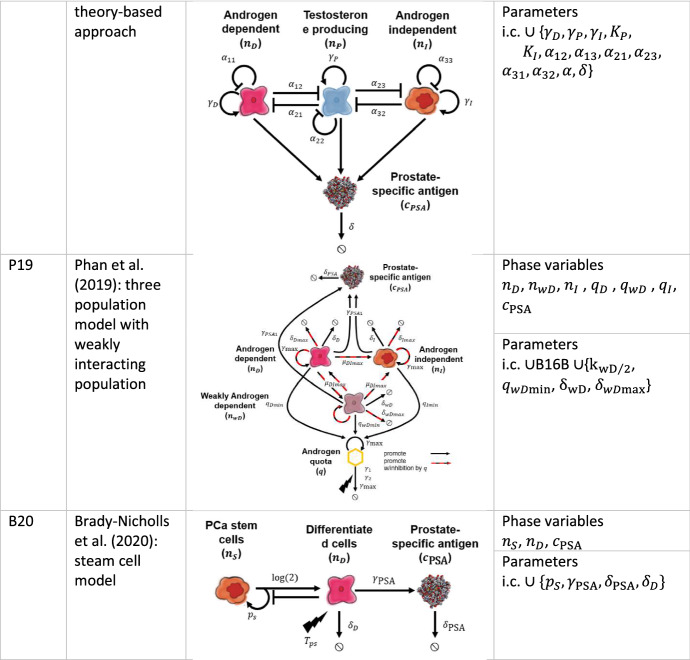
Model compartment sketches with phase variables and parameters modeled in the inference process

Initial condition (i.c.) are not reported, but phase variables are. $\alpha = \big\{ k_{{\textit{DI}}/2} ,k_{ID/2} ,k_{q/2},q_{\max }$, $v_{\max },\delta_{D} ,\delta_{q} ,\mu_{{{\textit{DI}}{\text{max}}}},\mu_{ID\max}\big\}$. Simbols used for the compartments are self-explanatory

The models analyzed herein synonymously use longitudinal PSA data to infer biological mechanisms underlying the observed PSA dynamics. PSA alone limited the potentiality of the presented approach and did not identify a single dominant model. Further information is necessary to simulate accurately and ultimately predict patient-specific PSA trajectories and the corresponding biological drivers of resistance. PSA alone might not be a helpful biomarker due to several dominant environmental factors outside the models’ scopes that influence its evolution under treatment. The use of PSA as a surrogate marker for prostate cancer burden is indeed controversial. Overexpression of the PCA3 gene obtained from the mRNA in urine samples is proposed to be more suited to monitoring the cancer evolution (Bussemakers et al. 1999, p. 3; Laxman et al. 2008; Neves et al. 2008; Hessels and Schalken 2009, p. 3; Borros 2009).

Two alternative directions might to improve our understanding of the PSA as a prostate cancer monitor biomarker. From one side, a deeper understanding of the connection between PSA and tumor burden throughout model investigation might present the opportunity for a new class of models. Recently, the role of immature blood vessels formed under angiogenesis cues has been investigated to decrypt the relation between an increased tumor burden contemporaneous to decreasing PSA concentration (Barnaby et al. 2021). Additionally, models that include both PSA and androgen concentrations might present some advantages in the future. The modest but significant evidence of the E16 model over the other models might indicate a more critical relevance of the dormancy whose biology and mathematics are worth undoubtedly deeper understanding.

Exploring PSA model probability distributions to disentangle responsive and resistant patient cohorts in a clinical setting could be investigated through cross-correlations with PCA3 biomarkers. Such cross-correlation would provide independent verification of the analytical findings herein that remain, for the moment, data-driven and, therefore, entirely dependent on the one dataset utilized in all discussed models.

Alternatively, PSA could be a perfect biomarker, but inter-patient heterogeneity in resistance mechanisms may disallow identifying a single model for all patients. Additionally, different resistance mechanisms may evolve in an individual patient, with their respective contribution to the observed response dynamics changing during therapy. More complex models and dynamic adaptive weighting of different variables, terms, and parameters may be necessary. Such models, however, would be non-identifiable with the presently available data. A close dialogue between biologists, statisticians, and mathematical and genitourinary oncologists may help identify which data should be collected in future clinical studies to help detangle the complex prostate cancer response dynamics to intermittent ADT.

While the Bayesian framework is an invaluable tool to estimate model parameters and fit model dynamics to clinical measurements, the goodness of a fit informs neither the reliability of the estimated parameters nor the likelihood of a model representing the data chosen for the valid biological reason. Relatively invariant PSA profiles can be obtained for a significant range in each parameter, as it is the case of a weakly sensitive—highly non-identifiable parameter. This fact is often omitted in the modeling literature, where the results are often presented without structural or practical identifiability analysis. Many of the herein discussed models have not demonstrated structural identifiability, hence jeopardizing the attempt to claim the inference’s practical identifiability herein. Nevertheless, we stress that a model’s value may also be found in its interpretative role (Enderling and Wolkenhauer 2021). The complexity of the mechanism involved in the biological responses to intermittent ADT can be captured correctly for a single patient but fail for others. *Therefore, the model comparison is not intended to* provide an absolute *ranking; instead,* it provides *an instrument to explore the different biological mechanisms implemented* in mathematical models in clinically observed treatment response and progression dynamics.

## Supplement

We have performed a sensitivity analysis for all the models included in the paper. However, as this analysis overlaps with the original papers’ work, we do not include those results here. Our sensitivity is motivated by: (1) to understand the dependence of our results on the parameters. For example, if we can claim the possibility to split between relapsing ($\Omega$) and not to relapse ($\neg \Omega$) patients by exploiting some specific model parameter combination, then the robustness of our result worth be investigated on the same parameter sensitivity to assign it the correct relevance and to evaluate its possibility to be applied to clinical tumor forecasting. (2) The technique implemented for the sensitivity analysis investigates the parameter’s sensitivity and the best-fit orbital integration, i.e., over the available longitudinal data. This approach enhances our understanding of *when* a particular $\Omega /\neg \Omega$ segregating technique is more useful during or off-treatment, with consequent indications on the role that a model splitting potentiality might or might not have (and when) on a per-patient base. (3) Continuous but not differentiable functions might need particular attention in the computation of the sensitivities because of their definition as the Jacobian matrix’s function. This approach represents a current research field often omitted in the mathematical oncology literature and is worth being brought to light.

Therefore, in what follows, we exploit the direct differential method (DDM) for sensitivity analysis (Gu and Wang 2013) to track the time dependence of the sensitivity $S_{ij} = \frac{{\partial x_{i} (t,\widehat{\bf{p}})}}{{\partial p_{j} }}$, where in general it is *x*_*i*_ = *c*_PSA_ and *p*_*j*_ the generic parameter dependent on the particular model in the exam. For a generic vector field $\frac{{\partial \bf{x}\left( {\bf{p};t} \right)}}{\partial t} = f\left( {\bf{x},\bf{p}} \right)$ with $x\left( {t_{0} ,\bf{p}} \right) = \bf{x}_{0}$ we couple the integration of the SoE defining the model with:

30 $$\frac{{\partial S_{ij} \left( {t,\hat{\bf{p}}} \right)}}{\partial t} = \frac{{\partial f_{i} \left( {x_{k} \left( {t,\hat{\bf{p}}} \right),\hat{\bf{p}}} \right)}}{{\partial x_{k} \left( {t,\hat{\bf{p}}} \right)}}S_{kj} \left( {t,\hat{\bf{p}}} \right) + \frac{{\partial f_{i} }}{{\partial p_{j} }}.$$

Generalized sensitivity (Stechlinski et al. 2018), based on the concept of generalized derivative for non-smooth *c*_PSA_ profiles (Clarke 1990) and used because of the loss of differentiability at the bifurcation points $T_{ps} = \left\{ {0,1} \right\}$ on the treatment parameter, has also been considered. We will not report the DDM analysis if not relevant to strength our specific results and we refer to the original model paper for general sensitivity analysis of the presented models.

## Footnotes

1 We use compact set notation here, e.g., $0 < t \in \left[ {t_{\min } ,t_{\max } } \right]$ means all the possible values of t, positive, between $t_{{{\text{min}}}}$ and $t_{{{\text{max}}}}$, i.e., $0 < t_{{{\text{min}}}} \le t \le t_{{{\text{max}}}}$. Open brackets will exclude the borders, e.g., soon after $\tau_{i} \subseteq \left] {t_{{{\text{min}}}} ,t_{{{\text{max}}}} } \right[$ means that the interval $\tau_{i}$, e.g., $\tau = \left[ {a,b} \right]$ is properly included between $t_{{{\text{min}}}}$ and $t_{{{\text{max}}}}$, but the limits of $t_{{{\text{min}}}}$ and $t_{{{\text{max}}}}$ are excluded: $t_{{{\text{min}}}} < \tau_{i} < t_{{{\text{max}}}}$. This allows us to work with the domain of existence of the indicator functions but arbitrarily truncate it at $t_{\min }$ or $t_{{{\text{max}}}}$.

2 Note that in our case the indicator function ${\bf{1}}_{{\tau_{i} }}$ is not-continuous scalar function, but traditionally indicated with bold characters even if it is not a matrix or a vector.

3 Note how, in general $t_{0D} \ne t_{0I}$ for what is seen in Sect. 2.2.

4 Note how we could consider the resulting SoE as function of the variable $c_{A}$ to reach a fully algebraic solution of the system by taking the ration of $\frac{{{\text{d}}n_{D} }}{{{\text{d}}c_{A} }}/\frac{{{\text{d}}n_{I} }}{{{\text{d}}c_{A} }}$. Nevertheless, it is more fruitful to look at the trend of Eq. (6) as obtained by the best fit procedure introduced in the next section.

5 Note on the statistical analysis: Details of statistical implementation are often omitted to focus the present work on the mathematical biology aspects pertinent to the work and the journal, but available upon request to the authors.

6 Due to the complexity of the model, analogous inference approximations to P12B have been used in this analysis.

7 Throughout algebraic manipulators as Mathematica or MAPLE it is possible to show that the system characterizing equation for *c*_PSA_ is algebraic of order 12, whose complete numeric roots investigation is beyond the scope of the present paper.

8 Note on the integrators: details of numerical integration are often omitted to focus the present work on the mathematical biology aspects pertinent to the work and the journal, but available upon request to the authors.
